# Antidiabetic Medicinal Plants Used in Democratic Republic of Congo: A Critical Review of Ethnopharmacology and Bioactivity Data

**DOI:** 10.3389/fphar.2021.757090

**Published:** 2021-10-27

**Authors:** Félicien Mushagalusa Kasali, Justin Ntokamunda Kadima, Emanuel L. Peter, Andrew G. Mtewa, Clement Olusoji Ajayi, Jonans Tusiimire, Casim Umba Tolo, Patrick Engeu Ogwang, Anke Weisheit, Amon Ganafa Agaba

**Affiliations:** ^1^ Pharm-Bio Technology and Traditional Medicine Center, Mbarara University of Science and Technology, Mbarara, Uganda; ^2^ Department of Pharmacy, Faculty of Pharmaceutical Sciences and Public Health, Official University of Bukavu, Bukavu, Democratic Republic of Congo; ^3^ Department of Pharmacy, Faculty of Medicine, Mbarara University of Science and Technology, Mbarara, Uganda; ^4^ Department of Pharmacology, School of Medicine and Pharmacy, University of Rwanda, Huye, Rwanda; ^5^ Department of Innovation, Technology Transfer and Commercialization, National Institute for Medical Research, Dar es Salaam, Tanzania; ^6^ Chemistry Section, Department of Applied Studies, Institute of Technology, Malawi University of Science and Technology, Limbe, Malawi; ^7^ Department of Pharmacognosy, Faculty of Pharmacy, Obafemi Awolowo University, Ile-Ife, Nigeria; ^8^ Department of Pharmacology and Therapeutics, Faculty of Medicine, Mbarara University of Science and Technology, Mbarara, Uganda

**Keywords:** antidiabetic plants, ethnopharmacology, phytochemicals, bioactivity, Democratic Republic of Congo

## Abstract

Several studies have been conducted and published on medicinal plants used to manage Diabetes Mellitus worldwide. It is of great interest to review available studies from a country or a region to resort to similarities/discrepancies and data quality. Here, we examined data related to ethnopharmacology and bioactivity of antidiabetic plants used in the Democratic Republic of Congo. Data were extracted from Google Scholar, Medline/PubMed, Scopus, ScienceDirect, the Wiley Online Library, Web of Science, and other documents focusing on ethnopharmacology, pharmacology, and phytochemistry antidiabetic plants used in the Democratic Republic of Congo from 2005 to September 2021. The Kew Botanic Royal Garden and Plants of the World Online web databases were consulted to verify the taxonomic information. CAMARADES checklist was used to assess the quality of animal studies and Jadad scores for clinical trials. In total, 213 plant species belonging to 72 botanical families were reported. Only one plant, *Droogmansia munamensis,* is typically native to the DRC flora; 117 species are growing in the DRC and neighboring countries; 31 species are either introduced from other regions, and 64 are not specified. Alongside the treatment of Diabetes, about 78.13% of plants have multiple therapeutic uses, depending on the study sites. Experimental studies explored the antidiabetic activity of 133 plants, mainly in mice, rats, guinea pigs, and rabbits. Several chemical classes of antidiabetic compounds isolated from 67 plant species have been documented. Rare phase II clinical trials have been conducted. Critical issues included poor quality methodological protocols, author name incorrectly written (16.16%) or absent (14.25%) or confused with a synonym (4.69%), family name revised (17.26%) or missing (1.10%), voucher number not available 336(92.05%), ecological information not reported (49.59%). Most plant species have been identified and authenticated (89.32%). Hundreds of plants are used to treat Diabetes by traditional healers in DRC. However, most plants are not exclusively native to the local flora and have multiple therapeutic uses. The analysis showed the scarcity or absence of high-quality, in-depth pharmacological studies. There is a need to conduct further studies of locally specific species to fill the gap before their introduction into the national pharmacopeia.

## 1 Introduction

Most African traditional healers who detain ancestral heritages are illiterate, and their knowledge transmitted verbally from generation to generation is at risk of disappearing. To minimize such risk, the World Health Organization (WHO) recommends scientists carry out ethnopharmacological and experimental studies to record folk knowledge, create databases, and validate scientifically traditional claims from the perspective of developing improved medications (WHO, 2013). WHO estimates that 80% of people rely on conventional medicine to meet primary health care needs, and most of them use remedies from plants (Surya et al., 2014). Ethnopharmacological surveys help gather holistic knowledge and practices of conventional healthcare systems. Experimental investigations evaluate efficacy and safety by developing suitable standardized pharmaceutical dosage forms that can complement, if not replace, current modern medicines. Medicinal plants used as complementary/alternative medicines (CAM) to manage various diseases provide a real opportunity in developed and developing societies. In this sense, herbal medications appear to offer readily available means of managing metabolic disorders by minimizing the risk of side effects and sometimes potentiating the treatment outcomes of modern drugs (Etxeberria et al., 2012). Medicinal plants are also used as food and contain several healthy dietary compounds. For example, some flavonoids interfere with metabolic events and play a crucial role in preventing and managing metabolic disorders through different pathways (Farzaei et al., 2019).

One of the most explored diseases is diabetes mellitus (DM). Over 800 plant species showing hypoglycemic activities can be essential sources for discovering and developing new types of antidiabetic molecules (Patel et al., 2012). The magnitude justifies this craze that Diabetes is gaining more and more globally, making it a severe public health problem. Not long ago, the disease was associated with industrialization. DM is no longer a disease of high-income countries but a global health pandemic. In 2013, according to the International Diabetes Federation, the global population of adults with both type-1(DMT1) and type-2(DMT2) was projected to increase from 382 million to 592 million by 2035, with DMT2 accounting for 90–95% of cases (Glezeva et al., 2017). In Africa, the number was expected to double from 14 million in 2015 to 34 million by 2040. With its continuous and rapid increase in its prevalence worldwide, it should be one of the leading causes of morbidity and mortality in the coming years (Glezeva et al., 2017).

Recent data show about 1.7 million people suffer from DM in the Democratic Republic of Congo (DRC), ranking fourth in the top ten countries by diabetes cases in Africa (Zhivov et al., 2015; Kasangana et al., 2018). Like other African countries, and not withdrawing modern medicines, 80% of people rely on traditional medicine to meet primary health care needs (Mahomoodally, 2013). Ethnopharmacological and pharmacological studies have been conducted globally; however, the related data are disparate and uncontrolled. A preliminary review reported vernacular names, parts used, and the formulation of 70 medicinal plants used to treat DM in DRC. A few phytoconstituents and antidiabetic mechanisms are also mentioned (Jacques et al., 2015).

This review aimed to describe what is known hitherto about ethnopharmacological, pharmacological, and clinical studies embracing medicinal plants used to manage DM in the traditional medicine of the DRC, to highlight which plants are native or introduced, how they are formulated and used, what valid experimental studies have been conducted in preclinical and clinical phases. A critical analysis is made to assess the quality of studies carried inside DRC and resort similarities/discrepancies with studies conducted outside.

**FIGURE 1:**
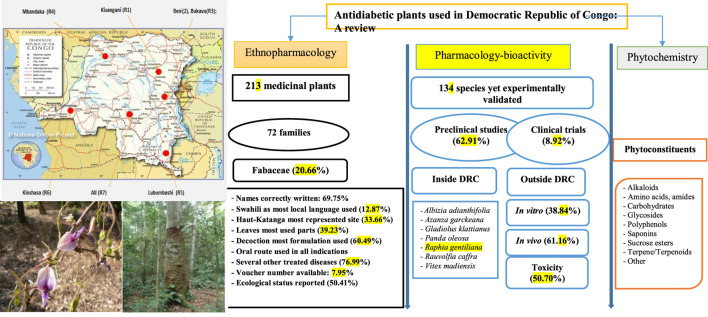
Graphical Abstract.

## 2 Methods

### 2.1 Literature Search Process

The review was an Internet search on Google Scholar, Medline, PubMed, Scopus, ScienceDirect, the Wiley Online Library, Web of Science, and other documents focusing on ethnopharmacology, pharmacology phytochemistry of antidiabetic plants used in the Democratic Republic of Congo from 2005 to September 2021. The review was conducted following Preferred Regulatory Items for Systematic Reviews and Meta-Analysis (PRISMA) guidelines 2009. A total of 34 studies were included. Ethnopharmacological/Ethnobotanical/Ethnomedicinal (*n* = 24), preclinical bioactivity (*n* = 9); and one clinical trial (*n* = 1) studies. One paper includes both an *in vivo* study and an Ethnobotanical survey.

**FIGURE 2:**
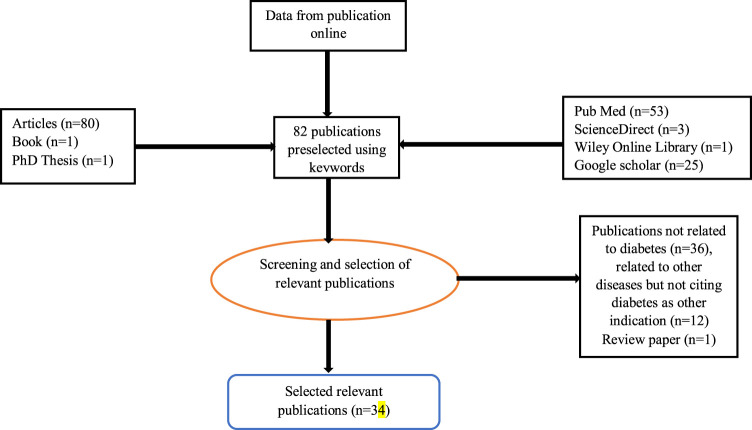
Flowchart for the selection of relevant publications.

### 2.2 Quality Critical Assessment

Studies that reported ethnopharmacology, phytochemistry, experimental pharmacology, and related clinical data were assessed for eligibility. The Kew Botanic Royal Garden and Plants of the World Online web databases were consulted to verify the taxonomic information on the species mentioned. All species names were checked at the UOB University herbarium. The quality of animal experiments reported was evaluated by examining the peer-reviewed publication, statement of control of temperature, appropriate animal model, compliance with animal welfare regulations for preclinical experiments, random allocation to treatment or management, blinded assessment of outcome, allocation sample size calculation, statement of potential conflict of interests, concealment, use of co-interventions/co-morbid. We used a CAMARADES checklist to assess the quality. Each task was given a quality score out of a possible total of 10 points. Thus, studies were categorized into low quality for mean score 1–5 and high quality for mean score 6–10 (Hooijmans et al., 2014; Auboire et al., 2018). The quality assessment of clinical trials has been evaluated using the Jadad scale for reporting randomized controlled trials based on randomization, blinding, withdrawals, and dropouts methods (Halpern and Douglas, 2007).

### 2.3 Statistical Values of Plant Species

Some indexes often express the frequency of quoting for botanical families and plant species. In the present review, the following indexes have been used: *Frequency of citation* (*FC* = Number of times a particular species was mentioned/Total number of times that all species were mentioned x 100); *Relative Frequency of Citation (RFC = FC/N; 0<RFC<1*): index, where FC is the number of informants who mentioned the use of the species and N, is the total number of informants (Tardío and Pardo-De-Santayana, 2008); *Use Value (UV = ƩU/n)* where U is the number of usable reports for a given plant species cited by each informant and n is the total number of informants interviewed for a given plant (Bano et al., 2014). The *Relative Importance Index (RII)* of each plant species was calculated based on the normalized number of pharmacological properties attributed to it and the normalized number of body systems (BS) it affects (Bennett and Prance, 2000).

### 2.4 Study Sites

**FIGURE 3:**
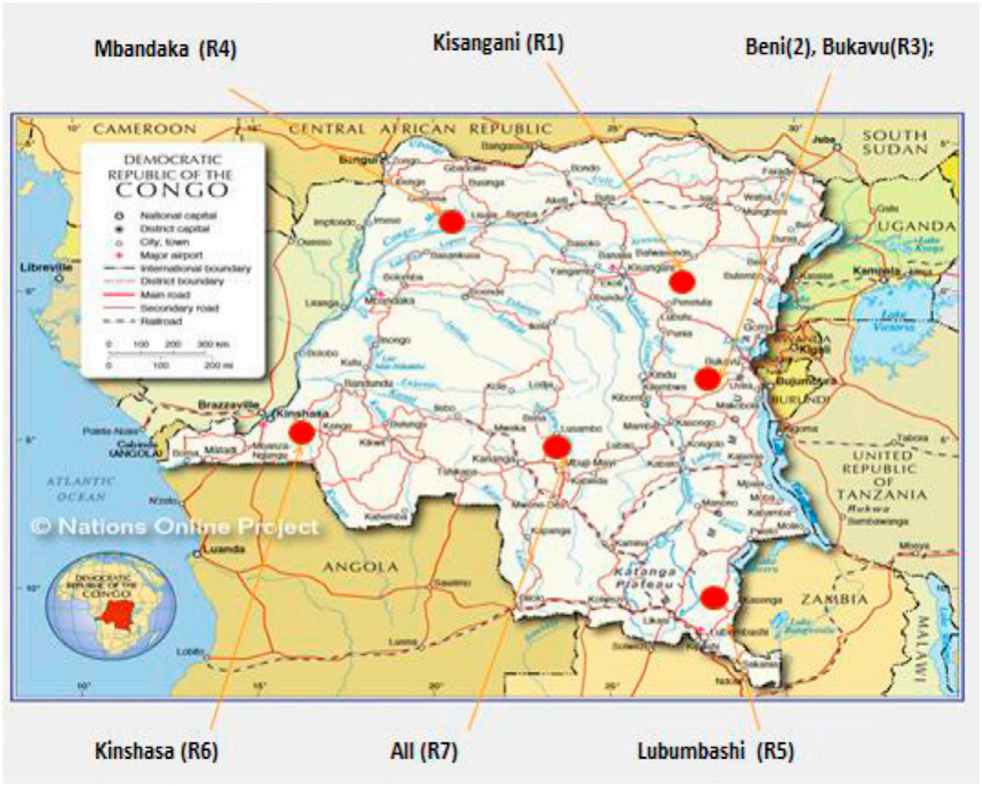
Study sites in the Democratic Republic of Congo.

Figure 3 shows different locations where 24 Ethnopharmacological/Ethnobotanical/Ethnomedicinal studies were conducted on the DRC map. The studies were done in Kisangani (R1) by Katemo et al. (2012), Mpiana et al. (2015); in Beni and Lubero (R2) by (Kasika et al., 2015); in Bukavu (R3) by (Karhagomba et al., 2013; Kasali et al., 2013, 2021; Chiribagula et al., 2020; Manya et al., 2020); in Mbandaka, Bagdolite, and Kungu (R4) by (Mongeke et al., 2018) ; in Lubumbashi, Kafubu, Kasumbalesa, Kipushi, Likasi and Sambwa (R5) by (Muya et al., 2014; Mbayo et al., 2016; Amuri et al., 2017, 2018; Bashige-Chiribagula et al., 2017; Mbuyi et al., 2019; Valentin et al., 2020) (Amuri et al., 2017); in Kinshasa, Kwango and Kongo central (R6) by (Ngbolua et al., 2016a, 2016b, 2019; Latham and Mbuta, 2017; Masunda et al., 2019; Pathy et al., 2021); in non-specified sites (R7) by (Moswa et al., 2005; Manzo, 2012).

## 3 Results

### 3.1 Ethnopharmacological Data

Table 1 describes the names, parts, forms used, locations, and some statistical values of plants cited. From 24 reviewed papers, we identified 213 plant species belonging to 72 botanical families.

**TABLE 1:** Ethnopharmacological specifications of plant species used to treat diabetes in DRC.

Family Scientific Name	Vernacular name	Part	Form	Site (References)	NC	FC	RFC	UV	RII
Acanthaceae									
*Brillantaisia patula* T.Anderson	Muleta (Zela), Lembalemba (Kongo),Lesongo (Swahili)	Sb	D	R5 Amuri et al. (2018)	1	0.0027	0.0008	0.0016	0.059
*Justicia flava* (Forssk.) Vahl	Luhe (Luba)	Sb	D	R5Amuri et al. (2018)	1	0.0027	0.0008	0.0016	0.059
Amaranthaceae									
*Chenopodium ambrosioides* L., *Dysphania ambrosioides* (L.) Mosyakin & Clemants (Synonym)	Kulamoka (Kongo), Dikanga (Tshiluba)	Wp	D	R6 Masunda et al. (2019) and R7 Moswa et al. (2005)	2	0.0054	0.0016	0.0008	0.029
Amaryllidaceae									
*Allium cepa* L.	Itunguru (Swahili) Ditungulu (Tshiluba)	Bk, Sd,Ap	D,M,I	R1 Katemo et al. (2012), R3 (Kasali et al. (2013), R5 Amuri et al. (2018); Mbuyi et al. (2019), R6 Masunda et al. (2019), and R7 Moswa et al. (2005)	6	0.0165	0.0047	0.0016	0.059
*Allium sativum* L.	Itungurusumu (Mashi), Hayi (Tshiluba)	Bk	D,P,F	R3 Kasali et al. (2013), R5 Amuri et al. (2018), R6 Masunda et al., (2019), and R7 Moswa et al., (2005)	4	0.0110	0.0032	0.0008	0.029
*Crinum ornatum* (Aiton) Herb.	Munsele bende (Kongo)	Lf	D	R1Katemo et al. (2012)	1	0.0027	0.0008	0.0008	0.029
Anacardiaceae									
Anacardium occidentale L.	Nkasu, diboto (Kongo)	Lf	N	R6 Latham and Mbuta (2017)	1	0.0027	0.0008	0.0016	0.059
*Mangifera indica* L.	Mutshiwa mangaya (Tshiluba), Hembe (Swahili), Mwembe (Mashi)	Sb, Ro,Lf	D	R1 Katemo et al. (2012), R3 Kasali et al. (2013); Chiribagula et al. (2020), R6 Masunda et al. (2019), and R7 Moswa et al. (2005)	5	0.0137	0.0039	0.0071	0.160
*Spondias mombin* L.	Mingenge (Not specified)	Lf	D	R6 Masunda et al. (2019)	1	0.0027	0.0008	0.0000	0.000
Anisophylleaceae									
*Anisophyllea boehmii* Engl.	Fungo (Sanga), Lufunga (Tabwa)	Ro	D	R5 Amuri et al. (2018)	1	0.0027	0.0008	0.0008	0.029
Annonaceae									
*Annona senegalensis* Pers. *Annona arenaria* Thonn (Synonym)	Kilolo (Kongo), Bomengo na esobe (Lingala), Lobo (not specified), Lomboloka (not specified)	Ro, Bk,Lf	D,N	R6 Ngbolua et al. (2016b); (2019); Latham and Mbuta (2017); Masunda et al. (2019); Pathy et al. (2021), and R7 (Moswa et al. (2005)	6	0.0165	0.0047	0.0110	0.229
*Monodora myristica* (Gaertn.) Dunal	Mpei (Lingala)	Fr, Sd	D	R6 Ngbolua et al. (2016a)	1	0.0027	0.0008	0.0016	0.059
*Xylopia aethiopica* (Dunal) A.Rich.	Nsombo (Not specified), Nkuya nkuya (Not specified)	Bb,Bk	D	R6 Masunda et al. (2019); Pathy et al. (2021)	2	0.0055	0.0016	0.0047	0.151
Apocynaceae									
*Catharanthus roseus* (L.) G.Don	Pervanche de Madagascar (French), Fulele (Ngwaka), Mtunda (Swahili)	Lf, Ro	D,M,N	R1 Katemo et al. (2012), R2 Kasika et al. (2015), R3 Kasali et al. (2013), R5 Amuri et al. (2018), R6 Latham and Mbuta (2017); Masunda et al. (2019), and R7 Moswa et al. (2005)	7	0.0192	0.0055	0.0063	0.183
*Diplorhynchus condylocarpon* (Müll.Arg.) Pichon	Mwenge (Swahili)	Ro	D	R5 Amuri et al. (2018)	1	0.0027	0.0008	0.0008	0.029
*Rauvolfia caffra* Sond.	Mutalala (Bemba)	Fr, Sb	D,M,N	R5 Amuri et al. (2017), (2018)	2	0.0055	0.0016	0.0008	0.029
*Rauvolfia obscura* K.Schum.	Mudisi (Kbla), Kilungu (Kongo)	Lf	D	R7 Moswa et al. (2005)	1	0.0027	0.0008	0.0000	0.000
*Rauvolfia vomitoria* Wennberg	Pandanganga (Luba)	Ro	D	R1 Katemo et al. (2012), R5 Amuri et al. (2018), and R6 Masunda et al. (2019)	3	0.0082	0.0024	0.0032	0.118
*Vinca minor* L.	Fololo (Lingala), Vinka nyeupe (Swahili)	Lf, Ro	D,M	R1 Katemo et al. (2012) and R3 Kasali et al. (2013)	2	0.0055	0.0016	0.0079	0.137
Arecaceae									
*Elaeis guineensis* Jacq.	Ba di ngasi (Kongo), Ngaji(Tshiluba), Palmier à huile (French)	Ro, Lt	D,N	R2 Kasika et al. (2015), R5 Amuri et al. (2018), and R6 Pathy et al. (2021)	3	0.0082	0.0024	0.0221	0.325
*Raphia gentiliana* De Wild.	Makeke (Not specified), BalempâBakulu (Lingala)	Lf, Sb	M	R6 Masunda et al. (2019)	1	0.0027	0.0008	0.0008	0.029
Aristolochiaceae									
*Aristolochia hockii* De Wild.	Kapanganganga (Bemba)	Ro	D	R5 Amuri et al. (2018)	1	0.0027	0.0008	0.0024	0.088
Asparagaceae									
*Asparagus africanus* Lam.	Mukoma wa kanyengelele (Luba)	Lf, Ro	D	R5 Amuri et al. (2018)	1	0.0027	0.0008	0.0016	0.059
Asphodelaceae									
*Aloe congolensis* De Wild. & T.Durand	Bà di nseki (not specified)	Lf	D	R6 Pathy et al. (2021)	1	0.0027	0.0008	0.0016	0.059
*Aloe vera* (L.) Burm.f.	Subiri (Swahili), Kizimia Muliro (Mashi)	Lf, Lt	M,Pr, P	R1 Katemo et al. (2012), R3 Kasali et al. (2013), and R5 Amuri et al. (2018)	3	0.0082	0.0024	0.0008	0.029
Asteraceae									
*Ageratum conyzoides* L.	Mpala kasakula (Kongo),	Lf	D	R7 Moswa et al. (2005)	1	0.0027	0.0008	0.0000	0.000
*Artemisia absinthium* L.	Kanyambuba kalume (Mashi)	Lf, Sd	D	R3 Kasali et al. (2013)	1	0.0027	0.0008	0.0024	0.088
*Artemisia annua* L.	Armoise annuelle (French), Sweet Annie(English)	Lf, Sd	D	R3 Kasali et al. (2013)	1	0.0027	0.0008	0.0047	0.151
*Bidens pilosa* L.	Mpotayambwa (Luba), Kashisha (Swahili) Kokoyalimo (Lokele)	Lf, Sd, Ro	D	R1 Katemo et al. (2012), R3 Kasali et al. (2013), and R5 Amuri et al. (2018)	3	0.0082	0.0024	0.0055	0.206
*Calendula officinalis* L.	Mundudi ndudi (Not specified)	Bk	D	R6 Masunda et al. (2019)	1	0.0027	0.0008	0.0000	0.000
Crassocephalum picridifolium (DC.)	Cifula (Mashi), Bupamba (Kibembe), Anatta (Kibembe)	Lf	D	R3 Chiribagula et al. (2020)	1	0.0027	0.0008	0.0000	0.000
*Gymnanthemum coloratum* (Willd.) H.Rob. & B.Kahn	Kilulukunju (Not specified)	Lf	D	R6 Masunda et al. (2019)	1	0.0027	0.0008	0.0000	0.000
*Tithonia diversifolia* (Hemsl.) A.Gray.	Bilombalomba (Lélé), Mubirizi (Mashi); Mululuca (Bembe)	Lf	M,C	R3 Kasali et al. (2013) and R5 Amuri et al. (2018)	2	0.0055	0.0016	0.0055	0.127
*Vernonia amygdalina* Delile	Nyata sololo, Mukari kari (Kongo), Mubirizi (Mashi), Mukadi kadi (Kiyanzi), Mindudi mintenla (Kiyombe)	Lf	D	R1 Katemo et al. (2012), R3 Karhagomba et al. (2013); Kasali et al. (2013), R6 Masunda et al. (2019), and R7 Moswa et al. (2005)	5	0.0137	0.0039	0.0095	0.249
*Vernonia shirensis* Oliv. & Hiern.	Kilulukunja (Swahili)	Lf, Ro	D	R5 Amuri et al. (2018)	1	0.0027	0.0008	0.0039	0.095
Basellaceae									
*Basella alba* L.	Nderema (Mashi), Ndelema (Kilega), Epinard Indien (French)	Lf	D	R3 Kasali et al. (2013)	1	0.0027	0.0008	0.0016	0.033
Betulaceae									
*Betula pendula* Roth		Lf	I	R6 Masunda et al. (2019)	1	0.0027	0.0008	0.0000	0.000
Bignoniaceae									
*Kigelia africana* (Lam.) Benth.	Kivungu (Luba)	Sb	D	R5 Amuri et al. (2018)	1	0.0027	0.0008	0.0016	0.033
*Spathodea campanulata* P.Beauv.	Cifulula, Langalanga (Mashi), Mbalimbali (Swahili)	Sb	D	R3 Kasali et al. (2013)	1	0.0027	0.0008	0.0016	0.033
Brassicaceae									
*Brassica juncea* (L.) Czern.	Ndunda (Kisoko), Nkofi (Kongo), Chou vert (French)	Lf	D	R1 Katemo et al. (2012)	1	0.0027	0.0008	0.0008	0.029
*Brassica oleracea* L.	Chou (French), Shu (Swahili), Nkofi nkolula (Kongo)	Lf	D,I	R1 Katemo et al. (2012), R5 Amuri et al. (2018), and R6(Masunda et al. (2019)	3	0.0082	0.0024	0.0024	0.088
Bromeliaceae									
*Ananas comosus* (L.) Merr.	Nanasi (Swahili) Ananas (French), Cikaka (Tshiluba)	Fr	D	R5 Amuri et al. (2018)	1	0.0027	0.0008	0.0016	0.059
Burseraceae									
*Canarium schweinfurthii* Engl.	Mpashi (Bemba), Mpafu (Luba)	Lf	D	R5 Amuri et al. (2018)	1	0.0027	0.0008	0.0024	0.062
Cactaceae									
*Opuntia ficus-indica* (L.) Mill.	Cactus (French)	Lf	C	R5 Amuri et al. (2018)	1	0.0027	0.0008	0.0008	0.029
Caricaceae									
*Carica papaya* L.	Kipawo (Sanga), Papai (Swahili), Ipapayi (Mashi)	Lf, Fr, Ro	D,I,M	R3 Kasali et al. (2013), R5 Amuri et al. (2018); Mbuyi et al. (2019), and 6 Masunda et al. (2019)	4	0.0110	0.0032	0.0095	0.196
Celastraceae									
*Maytenus senegalensis* (Lam.) Exell	Tshingala mutshi (Luba)	Lf, Ro	D	R5 Amuri et al. (2018)	1	0.0027	0.0008	0.0016	0.059
Salacia pynaertii De Wild	Mbondi (Not specified)	Lf	Rw	R6 Pathy et al. (2021)	1	0.0027	0.0008	0.0000	0.000
Chry*sobalanaceae*									
Parinari capensis Harv.	Nsudi funi (Not specified)	Lf	D	R6 Pathy et al. (2021)	1	0.0027	0.0008	0.0008	0.029
Clusiaceae									
*Garcinia huillensis* Welw. ex Oliv.	Mungindu (Tchokwe), Kisima (Not specified)	Ro, Lf, Fr	D,P	R5 Amuri et al. (2018) and R6 Pathy et al. (2021)	2	0.0055	0.0016	0.0063	0.157
*Garcinia kola* Heckel	Ngadiadia (Not specified)	Sd	D,N	R6 Ngbolua et al. (2016b), (2019)	2	0.0055	0.0016	0.0032	0.092
Combretaceae									
*Combretum celastroides* Welw. ex M.A.Lawson	Lukondambo (Luba), Mwina kyulu (Sanga)	Lf, Sb	D	R5 Amuri et al. (2018)	1	0.0027	0.0008	0.0008	0.029
*Terminalia catappa* L.	Madame (Lingala), Kalanga ya Wazungu (Swahili)	Lf	D	R1 Katemo et al. (2012)	1	0.0027	0.0008	0.0000	0.000
*Terminalia chebula* Retz.	Madame (Not specified)	Lf	I	R6 Masunda et al. (2019)	1	0.0027	0.0008	0.0000	0.000
*Terminalia mollis* M.A.Lawson	Kianga (Hemba), Tshibangu Mutshi (Tshiluba)	Lf, Ro	D	R5 Amuri et al. (2018)	1	0.0027	0.0008	0.0032	0.092
Commelinaceae									
*Palisota schweinfurthii* C.B.Clarke	Mabongu-bongu (Kiyanzi), Bunda-bunda (Kongo)	Lf, Wp	D	R7 Moswa et al. (2005)	1	0.0027	0.0008	0.0000	0.000
Convolvulaceae									
*Ipomoea mauritiana* Jacq.	Not reported	Tb	D	R1 Katemo et al. (2012)	1	0.0027	0.0008	0.0000	0.000
*Ipomoea spathulata* Hallier f.	Mulapa (Sanga)	Lf	C	R5 Amuri et al. (2018)	1	0.0027	0.0008	0.0008	0.029
Costaceae									
Costus lucanusianus J.Braun & K.Schum.	Boso boso, musanga vulu, ngo n’keni (Kongo)	Lf	N	R6 Latham and Mbuta (2017)	1	0.0027	0.0008	0.0016	0.059
*Costus phyllocephalus* K.Schum	Mafulungu (Kongo), Musangala (Kimbala)	Lf	D	R6 Masunda et al. (2019) and R7 Moswa et al. (2005)	2	0.0055	0.0016	0.0008	0.029
Cucurbitaceae									
*Cucumis sativus* L.	Concombre (French)	Fr	F	R5 Amuri et al. (2018)	1	0.0027	0.0008	0.0000	0.000
*Momordica charantia* L.	Lumbusu (Not specified)	Lf, Fr	D,I	R6 Masunda et al. (2019) and R7 Manzo (2012)	2	0.0055	0.0016	0.0055	0.127
Cyperaceae									
*Cyperus alternifolius* R.Br.	Ndao (Luba), Nsaku (Kongo)	Sb	D	R5 Amuri et al. (2018)	1	0.0027	0.0008	0.0024	0.062
Dilleniaceae									
*Tetracera poggei* Gilg	Mudia-ngulungu (Tshiluba)	Lf	D	R1 (Katemo et al. (2012) and R7 Moswa et al. (2005)	2	0.0055	0.0016	0.0047	0.124
Dioscoreaceae									
*Dioscorea bulbifera* L.	Nsoko ngamba, kimasoko (Not specified)	Tb	D	R6 Pathy et al. (2021)	1	0.0027	0.0008	0.0000	0.000
Dioscorea dumetorum (Kunth) Pax	Nsemi nsemi, ngamba (Kongo), kiazi kikuu (Swahili)	Tb	D	R6 Latham and Mbuta (2017)	1	0.0027	0.0008	0.0000	0.000
*Dioscorea praehensilis* Benth.	Bandindi (Not specified)	Lf	D	R6 Masunda et al. (2019)	1	0.0027	0.0008	0.0000	0.000
Ebenaceae									
*Diospyros heudelotii* Hiern	Mulolo kongolo (Kyzi), Lufwa lu ndomba (Kongo)	Ro	D	R7 Moswa et al. (2005)	1	0.0027	0.0008	0.0000	0.000
Euphorbiaceae									
*Alchornea cordifolia* (Schumach. & Thonn.) Müll.Arg.	Ditokoto (Tshiluba) Mambunzila (Kongo)	Ro	D,N	R2 Kasika et al. (2015), R6 Masunda et al. (2019), and R7 Moswa et al. (2005)	3	0.0082	0.0024	0.0063	0.183
*Croton macrostachyus* Hochst. ex Delile	Mutara mutshi (Bemba)	Lf	D	R5 Mbayo et al. (2016); Amuri et al. (2018)	2	0.0055	0.0016	0.0024	0.062
*Euphorbia prostrata* Aiton	Kapalatonvitonvi (Bemba)	Wp	D	R5 Mbayo et al. (2016)	1	0.0027	0.0008	0.0047	0.124
*Jatropha curcas* L.	Mbono (Swahili), Kilembelembe (Luba)	Lf, Sd, Ro	D,M,P	R1 Katemo et al. (2012) and R5 Mbayo et al. (2016); Amuri et al. (2018)	3	0.0082	0.0024	0.0126	0.287
*Maprounea africana* Müll.Arg.	Kafulumume (Bemba), Kazembezembe (Luba)	Ro, Sb	D	R5 Mbayo et al. (2016); Amuri et al. (2018), R6 Masunda et al. (2019) and R7 Moswa et al. (2005)	4	0.0110	0.0032	0.0079	0.137
*Ricinus communis* L.	Lundimba ndimba (Luba), Mubalika (Bemba)	Lf, Ro	D	R5 Amuri et al. (2018)	1	0.0027	0.0008	0.0039	0.095
*Tetrorchidium didymostemon* (Baill.) Pax & K.Hoffm.	bosefo, didi (Kilulua)	Lf	D	R1 Mpiana et al. (2015)	1	0.0027	0.0008	0.0000	0.000
Fabaceae									
*Abrus precatorius* L.	Kansengulu kandindi (Tshiluba), Abrus (French)	Sb	P	R3 Karhagomba et al. (2013) and R7 Moswa et al. (2005)	2	0.0055	0.0016	0.0032	0.065
*Acacia karroo* Hayne	Munga (Luba), Mutonge (Sanga), Mugunga (Hemba)	Lf, Sb	D	R5Amuri et al. (2018)	1	0.0027	0.0008	0.0016	0.059
*Acacia polyacantha* Willd.	Kibimbo, hibomo (hemba), Kimungamunga (Luba), Kashia (Swahili), Irangi (Kihavu)	Rb,Lf	D,I	R3 Chiribagula et al. (2020) and R5 Bashige-Chiribagula et al. (2017); Mbuyi et al. (2019)	3	0.0082	0.0024	0.0071	0.213
*Afrormosia angolensis* (Baker) Harms	Mubanga (Bemba), Mubanga kyulu (Luba)	Ro,Sb	D	R5 Amuri et al. (2018); Mbuyi et al. (2019)	2	0.0055	0.0016	0.0008	0.029
*Albizia adianthifolia* (Schumach.) W.Wight	Mulu (Kongo), Kampetanzevu(Tshiluba) Murunda (Swahili), Kapentazovu (Bemba)	Lf, Ro	D,N	R5 Amuri et al. (2017), (2018); Bashige-Chiribagula et al. (2017); Mbuyi et al. (2019); Valentin et al. (2020), R6 Latham and Mbuta (2017); Masunda et al. (2019), and R7 Moswa et al. (2005)	8	0.0220	0.0063	0.0055	0.154
*Albizia grandibracteata* Taub.	Mushebeye (Mashi) Kahunda (Kibembe)	Sb	D	R3 Kasali et al. (2013)	1	0.0027	0.0008	0.0024	0.088
*Arachis hypogaea* L.	Mwema (Bembe) Nguba (Lingala), kalanga (Swahili)	Lf	D	R5 Amuri et al. (2018)	1	0.0027	0.0008	0.0024	0.088
*Caesalpinia bonduc* (L.) Roxb.	Not reported	Lf	D	R6 Masunda et al. (2019)	1	0.0027	0.0008	0.0000	0.000
*Caesalpinia decapetala* (Roth) Alston	Lurhe (Mashi)	Lf	D	R3Kasali et al. (2013)	1	0.0027	0.0008	0.0024	0.062
*Cassia alata* L. *Senna alata* (L.) Roxb. (Synonym)	Lukunda bajanyi (Tshiluba), Mbaw-mbaw (Kongo)	Lf, Ro, Sd	D,M,N	R1 Katemo et al. (2012) and R6 Masunda et al. (2019)	2	0.0055	0.0016	0.0024	0.088
*Cassia occidentalis* L.	Lukunda bajanyi (Tshiluba), Mbaw-mbaw (Kongo), Mushigemanjoka (Mashi), Mujangajanga (Fuliru)	Lf, Ro, Sd	D,M,N	R1 Katemo et al. (2012), R3 Kasali et al. (2013); Chiribagula et al. (2020), R5 Amuri et al. (2017), (2018), and R7 Moswa et al. (2005)	6	0.0165	0.0047	0.0055	0.075
*Cassia petersiana* Bolle	Kafunga nashya (Bemba)	Ro	M	R5 Amuri et al. (2018)	1	0.0027	0.0008	0.0008	0.029
*Cassia sieberiana* DC.	Kandungandunga (Tshiluba), Mugunga (Hemba)	Lf	D,I,N	R5 Amuri et al. (2017), (2018)	2	0.0055	0.0008	0.0000	0.000
*Crotalaria spinosa* Hochst. ex Benth.	Kabalala (Sanga)	Ro, Sb	D	R5 Amuri et al. (2018)	1	0.0027	0.0008	0.0008	0.029
*Cyamopsis tetragonoloba* (L.) Taub.	Not reported	Lf	D	R6 Masunda et al. (2019)	1	0.0027	0.0008	0.0000	0.000
*Dalbergia boehmii* Taub.	Katembo mutshi (Luba), Katembo (Sanga)	Lf, Sb	D	R5 Amuri et al. (2018)	1	0.0027	0.0008	0.0039	0.095
*Droogmansia munamensis* De Wild.	Mununganunga (Bemba), Mulundeni (Lala)	Lf, Sb	D	R7 Amuri et al. (2018)	1	0.0027	0.0008	0.0008	0.029
*Eminia polyadenia* Hauman	-	Ro	M	R5 Muya et al. (2014)	1	0.0027	0.0008	0.0024	0.062
*Entada abyssinica* Steud. ex A.Rich.	Kipungu (Sanga)	Ro	D	R5 Amuri et al. (2018)	1	0.0027	0.0008	0.0032	0.065
*Erythrina abyssinica* Lam.	Kisongwa (Hemba), Katshiyitshiyi (Luba), Kikumbu, kikumbu ki nzambi (Kongo)	Ro,Lf,Bk	D,N	R5 Amuri et al. (2017), (2018), and R6 Latham and Mbuta (2017); Masunda et al. (2019)	4	0.0110	0.0032	0.0032	0.118
*Erythrophleum africanum* (Benth.) Harms	Kayimbi (Tshiluba)	Lf, Sb	D,M	R5 Amuri et al. (2018)	1	0.0027	0.0008	0.0016	0.059
*Glycine max* (L.) Merr.	Soja (Swahili)	Lf	D	R5 Amuri et al. (2018)	1	0.0027	0.0008	0.0000	0.000
*Indigofera arrecta* Hochst. ex A.Rich	Abwebwe (Kibembe), Musholotsi (Kihavu)	Ro	C	R3 Kasali et al. (2013)	1	0.0027	0.0008	0.0016	0.059
Indigofera capitata Kotschy	Nkeka za ngo (Not specified)	Wp	P	R6 Pathy et al. (2021)	1	0.0027	0.0008	0.0000	0.000
Isoberlinia tomentosa (Harms) Craib & Stapf	Mbaru (Mashi)	Lf	D	R3 Chiribagula et al. (2020)	1	0.0027	0.0008	0.0087	0.193
*Lonchocarpus katangensis* De Wild.	Chuya (Bemba)	Sb	M	R5 Amuri et al. (2018)	1	0.0027	0.0008	0.0016	0.059
*Millettia drastica* Welw. ex Baker	Mwengeti (Kongo), Nsiengieri (Kiyanzi)	Ro	D	R7 Moswa et al. (2005)	1	0.0027	0.0008	0.0039	0.068
*Millettia eetveldeana* (Micheli) Hauman	Mbwenge (Not specified)	Ro	D	R6 Masunda et al. (2019)	1	0.0027	0.0008	0.0000	0.000
*Millettia laurentii De Wild.*	Kiboto (Not specified)	Bk	D	R6 Masunda et al. (2019); Pathy et al. (2021)	2	0.0055	0.0016	0.0000	0.000
*Mucuna poggei* Taub.	Mpesa (Tshiluba)	Ro	D	R5 Amuri et al. (2018)	1	0.0027	0.0008	0.0000	0.000
*Pentaclethra macrophylla* Benth.	Mutie nzama (Kongo), Tshengesha (Tshiluba), Ngansi (Luba)	Sb	D	R7 Moswa et al. (2005)	1	0.0027	0.0008	0.0063	0.131
*Phaseolus lunatus* L.	Haricot (French), Maharagi (Swahili)	Lf, Ro	D,I	R5 Amuri et al. (2018)	1	0.0027	0.0008	0.0008	0.029
*Phaseolus vulgaris* L.	Cishimbo, mukenji (Mashi), Madesu (Lingala)	Gp	D,Tr	R3 Kasali et al. (2013), R5 Amuri et al. (2018), and R6 Masunda et al. (2019); Pathy et al. (2021)	4	0.0110	0.0032	0.0024	0.088
*Piliostigma thonningii* (Schumach.) Milne-Redh.	Kifumbe (Bemba, Luba)	Ro	M	R5Amuri et al. (2018)	1	0.0027	0.0008	0.0032	0.092
*Pterocarpus angolensis* DC.	Mukundambazu (Tabwa), Muyanga (Bemba), Sokosoko (Not specified)	Sb,Bk	D,P	R5 Amuri et al. (2018) and R6 Masunda et al. (2019); Pathy et al. (2021)	3	0.0082	0.0024	0.0039	0.095
*Pterocarpus marsupium* Roxb.	Nkila (Not specified)	Lf	D	R6 Masunda et al. (2019)	1	0.0027	0.0008	0.0000	0.000
*Pterocarpus tinctorius* Welw.	Mukula (Chokwe)	Ro	D	R5 Amuri et al. (2018)	1	0.0027	0.0008	0.0000	0.000
*Rhynchosia insignis* (O.Hoffm.) R.E.Fr.	Munkoyo (Swahili)	Ro	M	R5Amuri et al. (2018)	1	0.0027	0.0008	0.0008	0.029
*Scorodophloeus zenkeri* Harms	Kiwaya (Not specified)	Lf	D	R6 Masunda et al. (2019)	1	0.0027	0.0008	0.0000	0.000
*Senna timoriensis* (D.C.) H.S.Irwin & Barneby	Mapalata (Not specified)	Lf	D	R6 Masunda et al. (2019)	1	0.0027	0.0008	0.0008	0.029
*Swartzia madagascariensis* Desv.	Munienze (Luba), Mpampi (Tshiluba)	Ro	D	R5 Amuri et al. (2018)	1	0.0027	0.0008	0.0039	0.147
*Tephrosia vogelii* Hook.f.	Uleku (Kongo), Kai-kaya (Kybe), Bubawu (Tshiluba)	Lf	D	R7 Moswa et al. (2005)	1	0.0027	0.0008	0.0008	0.003
*Trigonella foenum-graecum* L.	Kiwaya (Not specified)	Lf	M	R6 Masunda et al. (2019)	1	0.0027	0.0008	0.0024	0.062
*Vigna sinensis* (L.) Savi ex Hausskn.	Lukunde (kikabinda)	Lf, Ro	D,M	R5 Amuri et al. (2018)	1	0.0027	0.0008	0.0000	0.000
Gnetaceae									
*Gnetum africanum* Welw	Fumbwa (Lingala)	Lf	D,P	R1 Katemo et al. (2012) and R6 Masunda et al. (2019); Pathy et al. (2021)	3	0.0082	0.0024	0.0000	0.000
Hypericaceae									
*Harungana madagascariensis* Lam. ex Poir.	Mukuta (Tshiluba), Kadwamuko (Mashi), Ndura (Swahili)	Lf, Ro, Sb	D	R3 Kasali et al. (2013), R5 Amuri et al. (2018), and R7 Moswa et al. (2005)	3	0.0082	0.0024	0.0079	0.190
Psorospermum corymbiferum Hochr.	Munkubagwa (Mashi)	Rb	M	R3 Chiribagula et al. (2020)	1	0.0027	0.0008	0.0055	0.127
Iridaceae									
*Gladiolus gregarius* Welw. Ex Baker	Litungulu ya zamba (Not specified)	Bb	D,N	R6 Ngbolua et al. (2019)	1	0.0027	0.0008	0.0008	0.029
*Gladiolus klattianus* Hutch	Kitala (Bemba), Kitokatoka (Luba)	Bk	D,M,N	R5 Amuri et al. (2017), (2018)	1	0.0027	0.0008	0.0016	0.059
*Coleus kilimandschari* Gürke	Mcubya (Bemba), Mutozo (Mashi), Mulavumba (Swahili)	Lf, Ro	D,I,M	R5 Amuri et al. (2018)	1	0.0027	0.0008	0.0047	0.124
Leucas martinicensis (Jacq.) R.Br.	Kanyamafundwe (Mashi), Namafundo (Fuliru)	Wp	D	R3 Chiribagula et al. (2020)	1	0.0027	0.0008	0.0039	0.095
*Ocimum gratissimum* L.	Malumba-lumba (Luba), Dinsusu-nsunsu (Kongo), Kitungu (Swahili), mayuyu (Kiyanzi), Dikondi, mazulu (Not specified)	Lf, Ro	D,I	R6 Masunda et al. (2019); Pathy et al. (2021), and R7 Moswa et al. (2005); Manzo (2012)	4	0.0110	0.0032	0.0150	0.271
*Ocimum minimum* L.	Dinsunsu nsusu Difioti (Not specified)	Lf	I	R6 Masunda et al. (2019)	1	0.0027	0.0008	0.0000	0.000
*Salvia officinalis* L.	Sauge (French)	Lf	I	R3 Kasali et al. (2013)	1	0.0027	0.0008	0.0039	0.095
*Vitex madiensis* Oliv.	Mufutu (Luba)	Lf, Ro	D,N	R5 Amuri et al. (2017), (2018), R6 Masunda et al. (2019), and R7 Moswa et al. (2005)	4	0.0110	0.0032	0.0047	0.124
Lauraceae									
*Persea americana* Mill.	Ikipapai (Lamba), Avocatier (French), Ivoka (Mashi)	Lf, Sb, Fr	D	R1 Katemo et al. (2012), R3 Kasali et al. (2013), R5 Amuri et al. (2018), and R6 Ngbolua et al. (2016a); Masunda et al. (2019)	5	0.0137	0.0039	0.0047	
Loganiaceae									
*Strychnos cocculoides* Baker.	Katongatonga (Luba), Bukoke (Hemba), Nzanza (Bemba)	Ro,Lf	D,I	R5 Amuri et al. (2018); Valentin et al. (2020)	2	0.0055	0.0016	0.0032	0.065
*Strychnos innocua* Delile.	Kakomekone (Swahili)	Ro	D	R5 Amuri et al. (2018)	1	0.0027	0.0008	0.0024	0.062
*Strychnos spinosa* Lam.	Kisongole (Bemba), Nsansa (Swahili)	Ro, Sb	D,N	R5 Amuri et al. (2017), (2018)	2	0.0055	0.0016	0.0047	0.098
*Strychnos stuhlmannii* Gilg.	Mubanga Kyilu (Bemba), Nkanga kyulu (Zela)	Ro	D	R5 Amuri et al. (2018); Valentin et al. (2020)	2	0.0055	0.0016	0.0016	0.059
Lythraceae									
*Punica granatum* L.	Not reported	Fw	I	R6 Masunda et al. (2019)	1	0.0027	0.0008	0.0000	0.000
Malvaceae									
*Adansonia digitata* L.	Mululu punga (Bemba)	Sb	D	R5 Amuri et al. (2018)	1	0.0027	0.0008	0.0008	0.029
*Azanza garckeana* (F. Hoffm.) Excell & Hillc	Muti ya makamashi (Swahili)	Lf, Sb	D,I,N	R5 Amuri et al. (2017), (2018)	2	0.0055	0.0016	0.0016	0.059
*Cola acuminata* (P. Beauv.) Schott & Endl.	Makasu (Not specified)	Lf, Sd	D,N	R6 Masunda et al. (2019); Ngbolua et al. (2019)	2	0.0055	0.0016	0.0008	0.029
*Cola nitida* (Vent.) Schott & Endl.	Mapio (Bambenga)	Fr	N	R4 Mongeke et al. (2018)	1	0.0027	0.0008	0.0008	0.029
*Grewia flava* DC.	Bungwe (Luba)	Lf, Sb	D	R5 Amuri et al. (2018)	1	0.0027	0.0008	0.0008	0.029
*Hibiscus esculentus* L., *Abelmoschus esculentus* (L.) Moench (Synonym)	Dongodongo (Lingala)	Fr	D,M,P	R1 Katemo et al. (2012) and R6 Masunda et al. (2019); Pathy et al. (2021)	3	0.0082	0.0024	0.0016	0.033
*Sida acuta* Burm.f.	Mudundu (Mashi)	Sb	D,N	R2 Kasika et al. (2015) and R3 Kasali et al. (2013)	2	0.0055	0.0016	0.0063	0.131
*Urena lobata* L.	Pungala (Not specified), Mpungala (Not specified)	Lf, Ro,Bb	D	R6 Masunda et al. (2019); Pathy et al. (2021)	2	0.0055	0.0016	0.0000	0.000
Meliaceae									
*Azadirachta indica* A.Juss	Nime (Not specified)	Lf	D	R6 Masunda et al. (2019)	1	0.0027	0.0008	0.0016	0.059
Menispermaceae									
*Penianthus longifolius* Miers	Not reported	Sb	M	R1 Katemo et al. (2012)	1	0.0027	0.0008	0.0032	0.092
Moraceae									
*Ficus benghalensis* L.	Nsanda (Not specified)	Lf, Bk	I	R6 Masunda et al. (2019)	1	0.0027	0.0008	0.0000	0.000
*Ficus exasperata* Vahl	Kikuya (Kongo)	Lf	D	R1 Katemo et al. (2012)	1	0.0027	0.0008	0.0032	0.065
*Ficus sycomorus* L.	Mukunyu (Swahili), Tshikuyi (Luba)	Lf, Sb, Ro	D	R5 Amuri et al. (2018)	1	0.0027	0.0008	0.0016	0.059
Moringaceae									
*Moringa oleifera* Lam	Moringa (Not specified), Mti maria (Mashi), Mlongelonge (Swahili)	Lf, Fw	I,Tr,D	R3 Kasali et al. (2013) and R6 Masunda et al. (2019); Pathy et al. (2021)	3	0.0082	0.0024	0.0032	0.065
Musaceae									
*Musa x sapientum* L.	Bananier (French)	Bb	D	R5 Amuri et al. (2018)	1	0.0027	0.0008	0.0032	0.092
Myrtaceae									
*Eucalyptus globulus* Labill.	Bikalubitus (Not specified)	Lf	I	R6 Masunda et al. (2019)	1	0.0027	0.0008	0.0024	0.062
*Psidium guajava* L.	Lipela (Swahili), Ngalafua (Tshiluba), Ngoyavi (Kongo)	Lf, Ro	D,M	R5 Amuri et al. (2018), R6 Masunda et al. (2019), and R7 Moswa et al. (2005)	3	0.0082	0.0024	0.0039	0.068
*Syzygium cumini* (L.) Skeels	Telezia (Swahili)	Fr	D	R1 Katemo et al. (2012) and R6 (Masunda et al. (2019)	2	0.0055	0.0016	0.0000	0.000
*Syzygium guineense* (Willd.) DC.	Musanfwa (Bemba)	Sb	D	R5 Amuri et al. (2018)	1	0.0027	0.0008	0.0032	0.092
Nyctaginaceae									
*Bougainvillea spectabilis* Willd	Bougainvillé (French)	Fw	M	R5 Amuri et al. (2018)	1	0.0027	0.0008	0.0008	0.029
Ochnaceae									
*Ochna schweinfurthiana* F.Hoffm.	Not reported	Ro	M	R5 Muya et al. (2014)	1	0.0027	0.0008	0.0024	0.062
Olacaceae									
*Olax obtusifolia* De Wild.	Kulokumo (Bemba)	Ro	D	R5Amuri et al. (2018)	1	0.0027	0.0008	0.0008	0.029
Oleaceae									
*Olea europaea* L.	Olivier (French)	Lf	I	R6 Masunda et al. (2019)	1	0.0027	0.0008	0.0000	0.000
Pandaceae									
*Panda oleosa* Pierre	Okali (Lingala)	Sb	D	R1 Katemo et al. (2012)	1	0.0027	0.0008	0.0008	0.029
Passifloraceae									
*Adenia gummifera* (Harv.) Harms	Komboponoke (Lamba), Kimboyi (Lala)	Sb	I	R5 Amuri et al. (2018)	1	0.0027	0.0008	0.0016	0.059
*Adenia venenata* Forssk.	Mafula (Luba)	Lf, Ro	D	R5 Amuri et al. (2018)	1	0.0027	0.0008	0.0000	0.000
Pedaliaceae									
*Sesamum angolense* Welw.	Kipalabwengo (Bemba)	Ro	D	R5 Amuri et al. (2018)	1	0.0027	0.0008	0.0000	0.000
*Sesamum indicum* L.	Wangila (Not specified)	Sd	D	R6 Masunda et al. (2019)	1	0.0027	0.0008	0.0000	0.000
Phyllanthaceae									
*Antidesma membranaceum* Müll.Arg. *Antidesma meiocarpum* J.Léonard (Synonym)	Tshilumba mutshi (Tshiluba), Mulambabwato (Bemba)	Lf, Sb	D,I	R5 Mbayo et al. (2016)	1	0.0027	0.0008	0.0024	0.062
*Antidesma venosum* E.Mey. ex Tul.	Kifubia (Luba), Misengo (Kongo), Nalushushwa (Fuliru)	Ro, Sb,Lf	D	R3 Manya et al. (2020) and R5 (Mbayo et al., (2016); Amuri et al. (2018)	3	0.0082	0.0024	0.0142	0.189
*Bridelia ferruginea* Benth	Kimwindu ki nseke (Kongo), Kindundu (Kintandu), Kimwindu (not specified)	Sb, Ro	D	R6 Masunda et al. (2019); Pathy et al. (2021) and R7 Moswa et al. (2005)	3	0.0082	0.0024	0.0118	0.232
*Hymenocardia acida* Tul.	Kapembe (Bemba), Lupep (Tchokwe), Kigeti (Kongo)	Ro	D	R5 Amuri et al. (2018) and R7 Moswa et al. (2005)	2	0.0055	0.0016	0.0063	0.157
*Phyllanthus amarus* Schumach. & Thonn.	Not reported	Lf	I	R6 Masunda et al. (2019)	1	0.0027	0.0008	0.0000	0.000
*Phyllanthus muellerianus* (Kuntze) Exell	Mupetwalupe (Bemba), Lulembalemba, Ludimba, lundimba, Kajimbajimba lujimba (Luba), Lulembalemba, Mulembalemba (Hemba)	Lf, Ro,Fr	D,Rw	R5 Mbayo et al. (2016); Bashige-Chiribagula et al. (2017); Mbuyi et al. (2019)	3	0.0082	0.0024	0.0102	0.173
*Phyllanthus niruri* L.	Kahungahunga (Tshiluba), Kapondo (Songye)	Wp	D	R5 Mbayo et al. (2016) and R6 Masunda et al. (2019)	2	0.0055	0.0016	0.0079	0.163
*Pseudolachnostylis maprouneifolia* Pax.	Musangati (Swahili), Musangali (Bemba)	Lf, Ro, Sd	D,C	R5 Mbayo et al. (2016); Amuri et al. (2018)	2	0.0055	0.0016	0.0102	0.173
*Uapaca kirkiana* Müll.Arg.	Masuku (Bemba, Luba)	Sb	D	R5 Mbayo et al. (2016); Amuri et al. (2018)	2	0.0055	0.0016	0.0079	0.163
Piperaceae									
*Piper guineense* Schumach. & Thonn.	Kapindi (Kongo), Ketshu (Luba), Nketu (Tshiluba)	Fr	P	R3 Kasali et al. (2013) and R7 Moswa et al. (2005)	2	0.0055	0.0016	0.0055	0.101
Poaceae									
*Cymbopogon citratus* (DC.) Stapf	Majani tshai (Swahili), Sinda (Kongo), Citronelle (French), Lemongrass (English)	Lf	D	R1 Katemo et al. (2012), R3 Karhagomba et al. (2013), and R6 Masunda et al. (2019)	3	0.0082	0.0024	0.0024	0.062
*Cymbopogon densiflorus* (Steud.) Stapf	Lusangu sangu (Not specified)	Lf	I,D	R6 Latham and Mbuta (2017); Masunda et al. (2019)	2	0.0055	0.0016	0.0071	0.160
*Oryza sativa* L.	Loso (Not specified)	Lf	D	R6 Masunda et al. (2019)	1	0.0027	0.0008	0.0000	0.000
*Zea mays* L.	Muyindi (Swahili), Cigonji (Mashi)	Sp	D	R3 Kasali et al. (2013)	1	0.0027	0.0008	0.0055	0.180
Polygalaceae									
*Polygala acicularis* Oliv.	Lunsambi nsambi (Not specified)	Lf,Bk	D	R6 Masunda et al. (2019); Pathy et al. (2021)	2	0.0055	0.0016	0.0016	0.059
Proteaceae									
*Faurea saligna* Harv.	Mulemu (Sanga)	Ro	D	R5 Amuri et al. (2018)	1	0.0027	0.0008	0.0000	0.000
*Protea obtusifolia* Engl.	Mwinkala nikata (Tabwa)	Ro, Sb	D	R5 Amuri et al. (2018)	1	0.0027	0.0008	0.0000	0.000
Rhamnaceae									
*Maesopsis eminii* Engl.	Ndunga (Luba)	Lf, Sb	D	R5 Amuri et al. (2018)	1	0.0027	0.0008	0.0024	0.088
*Ziziphus mucronata* Willd.	Kankona (Luba, Bemba, Sanga)	Ro, Sb	D	R5 Amuri et al. (2018)	1	0.0027	0.0008	0.0016	0.033
Rubiaceae									
*Crossopteryx febrifuga* (Afzel. ex G.Don) Benth.	Mutoshi (Tshiluba), Konsekonse (Bemba), Mvala (Kongo)	Lf, Ro	D,M	R5 Amuri et al. (2018) and R7 (Moswa et al. (2005)	2	0.0055	0.0016	0.0032	0.065
*Mitragyna stipulosa* (DC.) Kuntze Hallea stipulosa (DC.) J.-F.Leroy (Synonym)	Liluku (Lingala), Tshindubula, Mutoshi (Tshiluba), Longwa, nlongu (Kongo),	Sb,Bk	D	R6 Latham and Mbuta (2017) and R7 Moswa et al. (2005)	2	0.0055	0.0016	0.0008	0.029
*Morinda citrifolia* L.	Nsiki (Not specified)	Bk	D	R7 Manzo (2012)	1	0.0027	0.0008	0.0024	0.062
*Morinda lucida* Benth	Nsiki (Kongo), Indombe (Lingala), Isuku (Swahili)	Lf, Sb	D,M	R1 Katemo et al. (2012), R6 Masunda et al. (2019), and R7 (Moswa et al. (2005)	3	0.0082	0.0024	0.0079	0.137
*Morinda morindoides* (Baker) Milne-Redh.	Kileso nkama (Kongo), Nkonga bululu (Tshiluba), Kongo bololo (Not specified)	Lf	D	R1 Katemo et al. (2012), R6 Ngbolua et al. (2016b); Masunda et al. (2019); Pathy et al. (2021), and R7 Moswa et al. (2005)	5	0.0137	0.0039	0.0102	0.173
*Nauclea latifolia* Sm. *Sarcocephalus latifolius* (Sm.) E.A.Bruce (Synonym)	Lolo kienga (Kongo), Bungondo (Tshiluba)	Ro, Sd	D	R6 Ngbolua et al. (2016b), (2019); Masunda et al. (2019) and R7 Moswa et al. (2005)	4	0.0110	0.0032	0.0047	0.098
*Sarcocephalus pobeguinii* Hua ex Pobég*.*	Kenga kimansa (Not specified)	Lf	D,N	R6 Masunda et al. (2019)	1	0.0027	0.0008	0.0000	0.000
Rutaceae									
*Citrus limon* (L.) Osbeck	Citronier (French), Indimu (Mashi), Chunghwa kali (Swahili),	Fr, Ro	D,Pr	R1 Katemo et al. (2012), R3 Kasali et al. (2013), and R5 Amuri et al. (2018)	3	0.0082	0.0024	0.0095	0.249
*Citrus x aurantium* L., *Citrus sinensis* (L.) Osbeck.(Synonym)	N’lala (Kongo), Dingama (Kongo), Ndimu (Swahili), Lala (Kongo), Oranger doux(French)	Lf, Ro, Fr	D,M	R5 Amuri et al. (2018), R6 Latham and Mbuta (2017), and R7 (Moswa et al. (2005)	3	0.0082	0.0024	0.0047	0.177
*Zanthoxylum chalybeum* Engl.	Mpupwe kiulu (Luba), Pupwe (Bemba)	Lf, Sb, Ro	D	R5 Amuri et al. (2018)	1	0.0027	0.0008	0.0039	0.147
Santalaceae									
*Viscum album* L.	Not reported	Lf	I	R6 Masunda et al. (2019)	1	0.0027	0.0008	0.0000	0.000
Simaroubaceae									
*Quassia africana* (Baill.) Baill.	Mupeshipe (Not specified), Munkadi nkadi (Not specified)	Lf,Ro	M,D,N	R6 Ngbolua et al. (2019); Pathy et al. (2021)	2	0.0055	0.0016	0.0118	0.232
Solanaceae									
*Physalis angulata* L.	Ndimba, lumbundu (Not specified)	Wp, Lf, Fr	D	R7Manzo (2012)	1	0.0027	0.0008	0.0047	0.124
*Physalis peruviana* L.	Mbuma, Mpuhuhu (Mashi), Mbupuru (Kinande)	Lf	D	R3 Kasali et al. (2013)	1	0.0027	0.0008	0.0024	0.088
*Schwenckia americana* L.	Lunzila nzila, Yabala mbula, Tumpa di nkombo (Kongo)	Wp	D	R6 Masunda et al. (2019) and R7 (Moswa et al. (2005)	2	0.0055	0.0016	0.0087	0.166
*Solanum aethiopicum* L., *Solanum gilo* Raddi (Synonym), *Solanum subsessile* De Wild. (Synonym)	Nyanya (Swahili), Mutete (Luba)	Ro,Fr, Lf, Sd	D,F	R1 Katemo et al. (2012), R5 Amuri et al. (2018), and R6 Masunda et al. (2019)	3	0.0082	0.0024	0.0008	0.029
*Solanum americanum* Mill., *Solanum nigrum* L. (Synonym)	Makeke (Swahili), Mulunda (Mashi)	Lf	D	R1 Katemo et al. (2012) and R3 Kasali et al. (2013)	2	0.0055	0.0016	0.0032	0.092
*Solanum melongena* L.	Mbolongo (Not specified)	Fr	D	R6 Masunda et al. (2019)	1	0.0027	0.0008	0.0000	0.000
*Solanum seretii* De Wild.	Impwa (Bemba)	Ro	D	R5 Amuri et al. (2018)	1	0.0027	0.0008	0.0016	0.059
*Solanum tuberosum* L.	Pomme de terre (French), Birai (Swahili)	Tb	F	R5 Amuri et al. (2018)	1	0.0027	0.0008	0.0008	0.029
Thomandersiaceae									
*Thomandersia hensii* De Wild. & T.Durand	Ikoka (Turumbu), Liowa (Topoke)	Lf	D	R1 Katemo et al. (2012)	1	0.0027	0.0008	0.0071	0.186
Urticaceae									
*Musanga cecropioides* R.Br. ex Tedlie	Nsanga (Kongo), Mulombele (Tshiluba)	Lf	I	R7 Moswa et al. (2005)	1	0.0027	0.0008	0.0039	0.095
*Myrianthus arboreus* P.Beauv.	-	Ro	D	R6 Latham and Mbuta (2017)	1	0.0027	0.0008	0.0063	0.131
*Urtica dioica* L.	Chachingi (Mashi)	Lf	T,I	R3 Kasali et al. (2013)	1	0.0027	0.0008	0.0039	0.147
Verbenaceae									
*Lantana camara* L.	Mavi ya kuku (Swahili)	Lf	D,I	R5 Amuri et al. (2018)	1	0.0027	0.0008	0.0071	0.160
*Lippia multiflora* Moldenke	Bulukuti (Kongo), Filia m’filu filu (Kiyombe), Bulukutu, mbulunkutu (Kongo)	Lf	I,D,P	Latham and Mbuta (2017); Masunda et al. (2019); Pathy et al. (2021), and 7 Moswa et al. (2005)	4	0.0110	0.0032	0.0039	0.121
*Stachytarpheta indica* (L.) Vahl	Telezia (Swahili)	Lf	D	R3 Kasali et al. (2013)	1	0.0027	0.0008	0.0008	0.029
Vitaceae									
*Vitis vinifera* L.	Raisin (French)	Lf	D	R5 Amuri et al. (2018)	1	0.0027	0.0008	0.0000	0.000
Zingiberaceae									
*Aframomum melegueta* K. Schum.	Mundongo (Lingala), Ndungu zi nzo (Kongo)	Sd	P	R7 Moswa et al. (2005)	1	0.0027	0.0008	0.0024	0.088
*Zingiber officinale* Roscoe	Tangawisi (Swahili, Luba, Kongo), Nungu zikanda (Kybe), Tangawiwi (Lingala), Nunguzikanda (Kiyombe)	Rz	P	R7 Moswa et al. (2005)	1	0.0027	0.0008	0.0071	0.160
Zygophyllaceae									
*Balanites aegyptiaca* (L.) Delile	Mubambangoma (Swahili), Mbambangoma (Luba)	Ro	D	R5 Amuri et al. (2018)	1	0.0027	0.0016	0.0032	0.092

Legend: The parts: Ap(aerial part); Bb(bulb); Bk(bark); Fr(fruit); Fw(flower); Gp(green pods); Lf(leaf); Lt(Latex); Ro(root); Rb(Root bark); Rz(rhizome); Sd(seed); Sb(stem bark); Sp(spathe); Tb(tuber); and Wp(whole plant). Forms: D(decoction), I(infusion), M(maceration), T(Tincture), Tr(Trituration), N(Not specified), Pr(Pression), C(Chewing), P(Powder), Rw(Raw) Regions: R1(Kisangani), R2(Beni and Lubero), R3(Bukavu), R4(Bagdolite and Kungu), R5(Lubumbashi, Kafubu, Kasumbalesa, Kipushi, Likasi and Sambwa), R6(Kinshasa, Kwango and Kongo central). Quantitavive Ethnopharmacology: FC(Frequency of citation), NC(Number of citations), RFC(Relative Frequency of Citation), RII(Relative Imprtance Index), UV(Use value).

**FIGURE 4:**
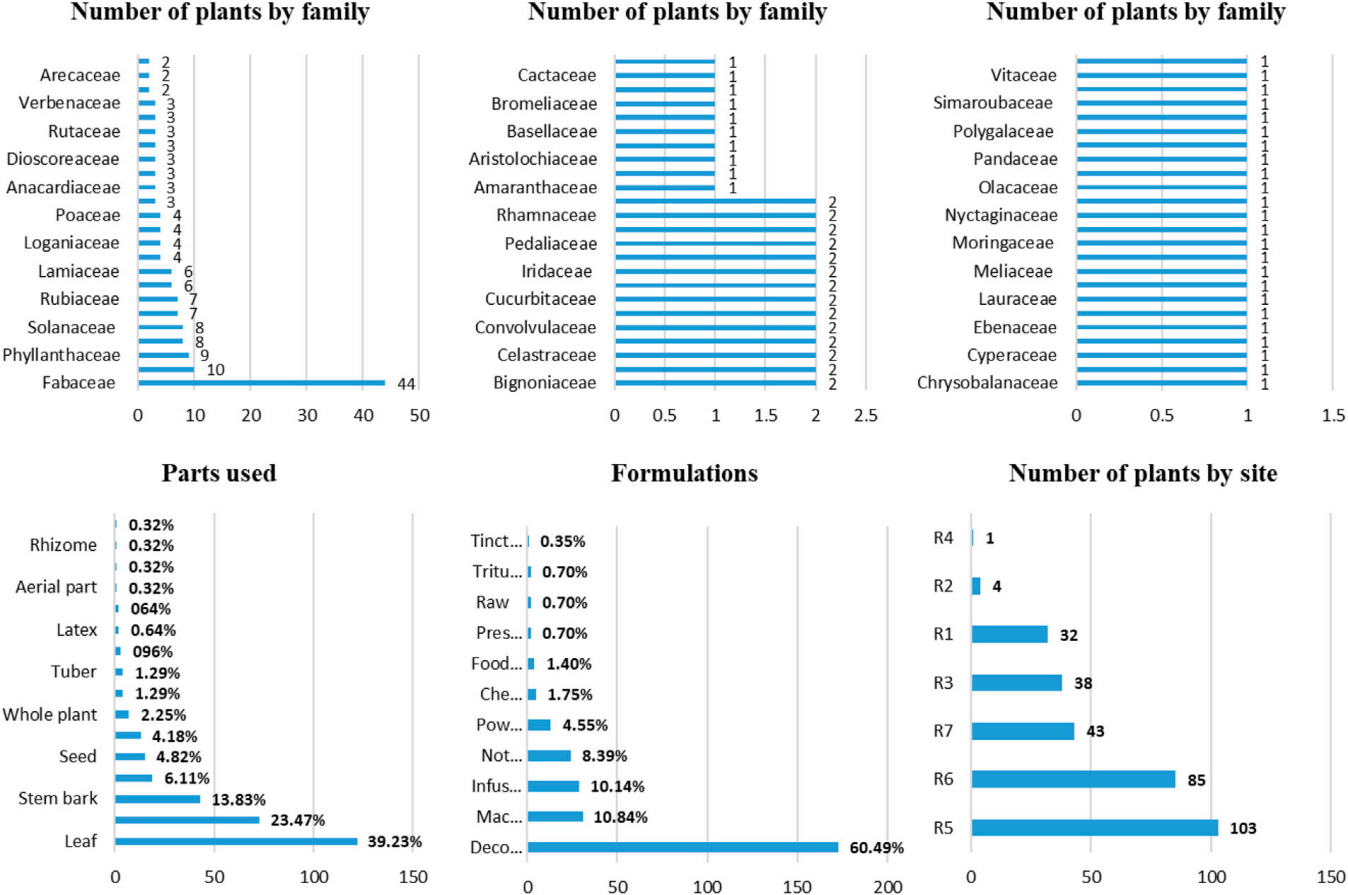
Frequencies of antidiabetic plants in DRC by botanical families, parts used, formulations, and sites.

As shown in Figure 4, the most frequent botanical families were Fabaceae with 44(20.66%) species, Asteraceae 10(4.69%), Phyllanthaceae 9(4.23%), Malvaceae 8(3.76%), Solanaceae 8(3.76%), Euphorbiaceae 7(3.29%), Rubiaceae 7(3.29%), Apocynaceae 6(2.82%), and Lamiaceae 6(2.82%). Most plants were found at the site R5(33.66%) and R6(27.78%). The distribution varied from study to study. *Catharanthus roseus* was found in almost all locations (6/7 sites) and *Allium cepa* in 5 zones. The vernacular names were linked or not to ethnic dialects. Swahili is the most reported language 48(12.87%), followed by Kongo 46(12.33%), Luba 36(9.65%), and Bemba 32(8.58%). In most cases, the vernacular name is not specified 47(12.60%) or not reported 8(2.14%). The formulations prepared consisted more often of decoction 173(60.49%), maceration 31(10.84%), and infusion 29(10.14%). The leaf is the most used part 122(39.23%), followed by roots 73(23.47%), and stem bark 43(13.83%).

### 3.2 Pharmacological Investigations Inside Democratic Republic of Congo

#### 3.2.1 Preclinical Pharmacological and Toxicological Investigations Inside Democratic Republic of Congo

**FIGURE 5:**
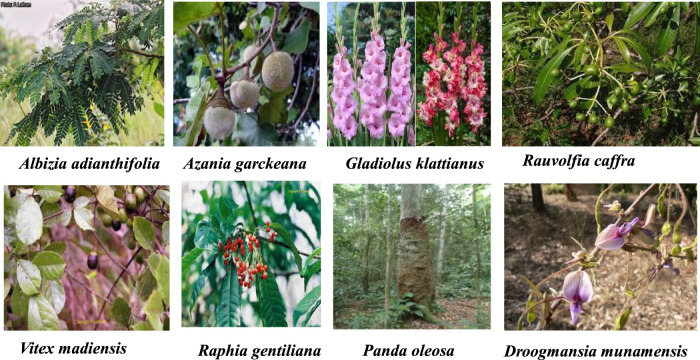
Plants that underwent experimental pharmacology inside DRC.

Only seven plants presented in Figure 5 were exclusively studied in experimental animals inside DRC; *Albizia adianthifolia*, *Azanza garckeana*, *Gladiolus klattianus*, *Panda oleosa*, *Raphia gentiliana*, *Rauvolfia caffra*, and *Vitex madiensis*; five studied in DRC. It also reported the only plant species native exclusively from DRC.

#### 3.2.2 Preclinical Toxicological Investigations Inside Democratic Republic of Congo

Many toxicological studies have been carried out in animals (rodents) using plant extracts. Some studies have been undertaken in mice, guinea pigs, and rabbits to explore the acute toxicity of *Panda oleosa*. Endpoints consisted mainly of mortality, pathophysiological syndromes, and microscopic examination of the pancreas and other vital organs pathological changes. The sub-chronic evaluation focused on assessing biochemical, hematological, and histopathological markers after a relatively long period (14 days and sometimes 90 days). The level of exposure to different organs, including the fetus, liver, kidney, heart, etc., of different doses of plant extracts was also determined. Thus, most plant extracts produce a toxic effect in specific organs or systems at high doses.

#### 3.2.3 Clinical Trials Inside Democratic Republic of Congo

Data from the present study showed the lack of local clinical trials of antidiabetic plants used to manage Diabetes in the DRC. Of seven native herbals, only *Raphia gentiliana* fruit extract was given to 25 males and 20 females, aged 18–50 years old, with normal blood sugar levels (Mpiana et al., 2013). Thirty persons were submitted to the fruits of *R. gentiliana* as food (0.14 g/kg), while fifteen were introduced to the glucose solution (0.07 g/kg) (standard). The glycemia was measured by spectrophotometry, and the triangle surface area ratio method was used to calculate the glycemic and load index. The observed values of glycemic index and load were −3.1% and −1.36%. The approach followed by the authors did not comply with any clinical trial requirements, and instead, their behavior went like traditional healers themselves.

### 3.3 Phytochemical Investigations

Some studies have been undertaken to explore the chemical composition of *Panda oleosa, Physalis peruviana*, and *Vernonia amygdalina*.

## 4 Discussion

### 4.1 Ethnopharmacological Data

#### 4.1.1 Ethnobotanical Information Reported

The analysis presented in Table 2 showed that the ecological status was reported for 185(50.41%) plants and not for 182(49.59%). On the other hand, plant identification was reported in 326(89.32%) cases and not 39(10.68%).

**TABLE 2:** Quality analysis of ethnobotany information.

Ecological source	%	Errors detected	*%*
Ecological status reported	50.41	Author name correct	69.59
Ecological status not reported	49.59	Author name incorrect	16.16
Common plants to DRC and Africa	54.93	Author name absent	14.25
Introduced from the Americas and Europe	14.55	Family name unchanged	81.64
Exclusively native to DRC (*D. munamensis*)	0.47	Family name revised	17.23
Origin not mentioned	30.05	Family name absent	1.10
Voucher number reported	7.95	Plant name confused with its synonym	4.69
Voucher number not reported	92.05	Plant identification reported	89.32
		Plant identification not reported	10.68

The errors in plant authors included entirely different authors, spelling mistakes, inappropriate use of the period, improper use of bracket, and incomplete author name.

The origin of plants was specified in 69.95% and not in 30.05% of species. However, 54.93% of plants with known origin were native to Africa, 14.55% species were introduced, and *Droogmansia munamensis* was the only species exclusively native to DRC flora (“Haut-Katanga”). Concerning the data quality, the author names of plant species were correctly written in 69.59% of cases, not correctly registered in 16.16%, or absent in 14.25%. Furthermore, 17.26% of plants had family names changed, and 81.64% not changed. In few cases (*n* = 10), the main plant was confused with its synonym. For example, *Antidesma metacarpus* (*A. membranaceum*)*, Annona senegalensis* (*A. arenaria*), *Cassia alata* (*Senna alata*), *Chenopodium ambrosioides* (*Dysphania ambrosioides*), *Citrus x aurantium* (*C. sinensis*), *Hibiscus esculentus* (*Abelmoschus esculentus*), *Mitragyna stipulosa* (*Hallea stipulosa*), *Nauclea latifolia* (*Sarcocephalus latifolius*), *Solanum americanum* (*S. nigrum*), and *Solanum gilo* (*S. aethiopicum* and *S. subsessile*). Most species (89.32%) from different sites were identified and authenticated in other herbariums or laboratories of ecology, but only a few (7.95%) had a voucher number published. This situation implicates the responsibilities of publishers and reviewers. Table 2 shows the quality analysis of findings compared to data from Plants of the World Online web (http://powo.science.kew.org) database and http://plantsoftheworldonline.org via the Royal Botanic Garden Kew database.

#### 4.1.2 Ethnopharmacological Data Reported

The country is home to different ethnic groups, making it one of the most diverse countries globally, with more than 200 other ethnic groups speaking an estimated 213 native languages. Sometimes referred to as the Baluba, the Luba people are the largest ethnic group. The community is native to the Kasai, Maniema, and Katanga regions. The Mongo people comprise several smaller constituent groups, including the Mbole, Ekonda, Boyela, Bolia, and Nkutu. The Kongo ethnic group is native to DRC and Angola, speaking Kongo alongside Lingala, Kyanzi, and Kintandu. The Mangbetu ethnicity is concentrated within the Orientale Province (Kisangani). The Zande people reside in the tropical rainforest and the savanna and speak nearly five dialects of the Azande language. The Pygmies are considered to have been some of the earliest peoples to inhabit the Congo River Basin. Their short stature characterizes them, they are mainly hunters and gatherers, and they occupy the rainforest. The plants are distributed within tropical and subtropical ecological regions, flooded grasslands, moist broad-leaf forests, savannas, and mangroves.

Swahili is the most reported language 48(12.87%), followed by Kongo 46(12.33%), Luba 36(9.65%), Bemba 32(8.58%), Tshiluba 29(7.77%), Mashi 26(6.97%), French 21(5.63%), and Lingala 14(3.75%). After le French, which is the official language, there are four regionally distributed national languages, including Ciluba (Tshiluba), Kongo, Lingala, and Swahili, among 213 native languages identified in DRC. Those four languages are used in out-group communication, in lower primary school years (mainly in rural and semi-urban areas), cultural and religious purposes, etc. (Kasanga, 2012).

Fabaceae was the most representative family, consistent with other studies that showed this family is commonly found in tropical rain and dry forests in the Americans and Africa (Burnham and Johnson, 2004). Around 60% of the Congo-basin lies in the DRC, the second-largest contiguous tract of tropical forests globally, and the greatest extent of tropical rainforests in Africa. It covers more than 100 million hectares (Abernethy et al., 2016).

The leaf was the most used part 122(39.23%), followed by roots 73(23.47%), and stem bark 43(13.83%). According to (Moshi et al., 2012), the frequent use of leaves is associated with ease of accessibility among the aboveground parts of plants in natural ecosystems. The formulations prepared consisted more often of decoction for 173(60.49%), followed by maceration 31(10.84%) and infusion 29(10.14%). However, in 24 cases (8.39%), the formulation has not been reported. Decoction has often been the effective formulation of herbal remedies as it is easy to prepare by mixing a drug with boiling water (Mahomoodally et al., 2016).

Out of 213 plants listed, 103(33.66%) were found at site R5 and 85(27.78%) at R6. The majority of plants had local vernacular names, except in few cases where the author did not mention the names. For instance, *Catharanthus roseus* was found at almost all locations (except site R4) and *Allium cepa* at five sites*.* However, *A. sativum, Cassia alata, C. occidentalis, Mangifera indica, Persea americana*, and *Vernonia amygdalina* were quoted at four locations.

#### 4.1.3 Validation of Ethnopharmacological Data

Studies undertook outside DRC confirmed the use of the majority of plants cited as antidiabetic remedies. *Albizia adianthifolia* was the most reported antidiabetic with eight citations representing an RFC of 0.0063, followed by *Catharanthus roseus* 7(RFC = 0.0055). However, *Allium cepa*, *Annona senegalensis*, and *Cassia occidentalis* were reported six times (RFC = 0.0047), followed by *Mangifera indica, Morinda morindoides*, *Phaseolus lunatus,* and *Vernonia amygdalina* with five citations (RFC = 0.0039).

Comparatively, *Elaeis guineensis* was endorsed by 28 uses; the (UV score of 0.0221 was the highest compared to *Ocimum gratissimum* (0.0150), *Antidesma membranaceum* (0.0142), *Jatropha curcas* (0.0126), *Bridelia ferruginea* (0.0118), and *Quassia africana* (0.0118). Also, *Balanites aegyptiaca*, which is employed to treat nine body systems, showed the highest Relative Importance Index (32.5%), compared to *Vitis vinifera* (28.7%), *Zingiber officinale* (27.1%), *Solanum seretii* (24.9%), *Thomandersia hensii* (24.9%), *Lippia multiflora* (23.2%) and *Stachytarpheta indica* (23.2%). The use-value indicators (Table 1) are relative and susceptible to changing since, in the methodology, many authors generally limit themselves to the total number of participants in the studies. The lack of information on the number of informants interviewed was commonly observed in the reviewed studies. According to the Declaration of Helsinki, the direct consequence is that it is no longer possible to analyze the quantitative aspects of these studies (The World Medical Association, 2001).

Consequently, it is not possible to quantify certain vital indexes such as the Cultural Importance Index (CII), Fidelity Level of Citation (FL), Family Use Value, Importance Consensus Factor (ICF), etc. It should be noted that some rare studies make an effort to investigate these parameters, although the information on the number of respondents remains a challenge. One of the weaknesses of ethnopharmacological surveys is that the respondents are often the healers themselves and not or seldom the users. The questionnaires do not scrutinize evidence on the number of people treated and outcomes.

Table 3 illustrates the information gathered through the literature for some plants that can manage Diabetes and other comorbidities and complications. Among the plants listed as antidiabetic, 164(76.99%) species are being used locally in the treatment of several other diseases, mainly infections (bacterial, parasitic, viral, fungal), gastrointestinal and abdominal disorders, cardiac and neurological diseases, gynecological disorders, sexual problems, wounds, dermatological, hematological and metabolic diseases. Commonly, no one plant holds only one indication due to the complexity of the chemical content. The data combine both inside and outside studies.

**TABLE 3:** Antidiabetic plants used locally for the treatment of other various disorders.

Disorders/effect	Plants used
Abdominal pain	*Afrormosia angolensis; Ageratum conyzoides; Allium sativum; Anisophyllea boehmii; Coleus kilimandschari; Cymbopogon densiflorus; Cyperus alternifolius; Dalbergia boehmii; Nauclea latifolia; Phaseolus lunatus; Psidium guajava; Solanum aethiopicum; Solanum seretii; Strychnos cocculoides; Strychnos spinosa; Uapaca kirkiana; Tithonia diversifolia; Ziziphus mucronata; Zingiber officinale; Pseudolachnostylis maprouneifolia; Maprounea africana; Acacia polyacantha*
Abdominal cramps	*Antidesma venosum; Cymbopogon densiflorus; Piper guineense*
Abortions repeated	*Musanga cecropioides; Antidesma venosum; Brillantaisia patula; Dalbergia boehmii; Schwenckia americana*
Abscess	*Antidesma venosum; Aloe vera; Annona senegalensis; Bidens pilosa; Chenopodium ambrosioides*
Amoebiasis	*Elaeis guineensis; Cassia occidentalis; Morinda lucida; Cymbopogon densiflorus; Morinda morindoides; Bridelia ferruginea; Caesalpinia decapetala; Carica papaya; Crossopteryx febrifuga; Garcinia huillensis; Hymenocardia acida; Garcinia kola; Harungana madagascariensis; Jatropha curcas; Justicia flava; Myrianthus arboreus; Alchornea cordifolia;* Psorospermum corymbiferum*; Pentaclethra macrophylla; Strychnos cocculoides; Tetracera poggei; Uapaca kirkiana; Tithonia diversifolia; Vinca minor; Vitex madiensis; Psidium guajava; Nauclea latifolia; Mangifera indica; Maprounea africana*
Anemia	*Annona senegalensis,* Isoberlinia tomentosa; *Phyllanthus muellerianus; Alchornea cordifolia; Hymenocardia acida; Ocimum gratissimum; Ficus sycomorus; Ochna schweinfurthiana; Persea americana; Piliostigma thonningii; Vitex madiensis; Momordica charantia*
Angina	*Coleus kilimandschari;* Isoberlinia tomentosa; *Morinda lucida*
Anorexia	*Ananas comosus; Tithonia diversifolia; Zingiber officinale*
Aphrodisiac	*Albizia adianthifolia; Antidesma venosum; Phyllanthus muellerianus; Uapaca kirkiana; Zingiber officinale*
Ascites	*Schwenckia americana; Xylopia aethiopica*
Asthenia	*Tithonia diversifolia*
Asthma	*Antidesma membranaceum; Catharanthus roseus; Cymbopogon densiflorus; Cyperus alternifolius; Elaeis guineensis; Lantana camara; Costus phyllocephalus; Ocimum gratissimum; Phyllanthus niruri; Schwenckia americana; Vitex madiensis*
Arthritis	*Allium cepa; Phaseolus vulgaris; Zea mays*
Backache	*Aframomum melegueta; Chenopodium ambrosioides; Cola acuminata; Gladiolus gregarious; Nauclea latifolia; Ocimum gratissimum; Zingiber officinale*
Birth troubles	*Adenia gummifera*
Bleunorrhagia	*Carica papaya; Citrus limon; Croton macrostachyus; Diplorhynchus condylocarpon; Ficus exasperata; Strychnos innocua; Strychnos spinosa; Tetracera poggei; Zingiber officinale*
Bronchitis	*Allium cepa*
Bronchopneumonia	*Ocimum gratissimum; Quassia Africana; Alchornea cordifolia*
Burns	*Aloe vera; Brassica oleracea*
Buruli ulcer	*Elaeis guineensis*
Cancer	*Brassica oleracea; Antidesma venosum; Catharanthus roseus; Chenopodium ambrosioides; Erythrina abyssinica; Erythrophleum africanum; Urtica dioica; Ageratum conyzoides; Aloe vera; Harungana madagascariensis; Zea mays; Vinca minor*
Cancer (prostate)	*Ageratum conyzoides*; *Arachis hypogaea; Bidens pilosa; Sida acuta*
Cataract Eye	*Moringa oleifera; Thomandersia hensii;* Crassocephalum picridifolium*; Euphorbia prostrata*
Chest pain	*Schwenckia americana*
Cholera	*Phyllanthus muellerianus*
Cold	*Cymbopogon densiflorus; Lantana camara; Morinda citrifolia, Ocimum gratissimum; Tithonia diversifolia*
Colitis	*Ageratum conyzoides, Carica papaya; Citrus limon; Morinda morindoides; Schwenckia americana; Vinca minor; Pseudolachnostylis maprouneifolia; Physalis peruviana; Mangifera indica*
Conjunctivitis	*Moringa oleifera; Mangifera indica*
Constipation	*Lantana camara; Ageratum conyzoides; Bridelia ferruginea; Carica papaya; Cassia occidentalis; Pentaclethra macrophylla; Persea Americana; Phyllanthus niruri; Jatropha curcas; Artemisia annua;* Leucas martinicensis; *Tithonia diversifolia; Rauvolfia vomitoria; Mangifera indica; Maprounea africana; Momordica charantia*
Convulsions	*Bridelia ferruginea; Vigna sinensis*
Cough	*Abrus precatorius*, *Aframomum melegueta*; *Aloe vera; Artemisia annua; Bidens pilosa; Carica papaya; Catharanthus roseus; Citrus limon; Citrus x aurantium; Coleus kilimandschari; Elaeis guineensis; Garcinia huillensis;* Isoberlinia tomentosa; *Jatropha curcas; Lantana camara; Myrianthus arboreus; Piliostigma thonningii; Zanthoxylum chalybeum; Zingiber officinale; Vitex madiensis; Piper guineense; Ocimum gratissimum; Lippia multiflora;* Crassocephalum picridifolium.
Delirium	*Ageratum conyzoides*
Dermatitis	*Abrus precatorius; Costus phyllocephalus*
Dehydration	Isoberlinia tomentosa
Diarrhea	*Cassia occidentalis; Balanites aegyptiaca; Annona senegalensis; Antidesma membranaceum; Bridelia ferruginea; Ficus exasperata; Ficus sycomorus;* Isoberlinia tomentosa; Leucas martinicensis; Psorospermum corymbiferum*; Persea americana; Sida acuta; Albizia adianthifolia; Dalbergia boehmii; Psidium guajava; Quassia Africana; Phyllanthus muellerianus; Acacia polyacantha; Antidesma venosum; Bidens pilosa; Phyllanthus niruri; Entada abyssinica; Syzygium guineense; Terminalia mollis; Uapaca kirkiana; Momordica charantia; Zea mays; Vinca minor; Pterocarpus angolensis; Piper guineense; Nauclea latifolia; Millettia drastica; Maytenus senegalensis*
Dizziness	*Vinca minor*
Dysentery	*Canarium schweinfurthii; Carica papaya; Droogmansia munamensis; Euphorbia prostrata; Strychnos cocculoides; Strychnos spinosa; Thomandersia hensii; Tetracera poggei; Uapaca kirkiana; Vernonia amygdalina; Xylopia aethiopica; Ziziphus mucronata; Psidium guajava; Pseudolachnostylis maprouneifolia*
Dysmenorrhea	*Aristolochia hockii; Artemisia absinthium; Carica papaya; Cassia alata; Balanites aegyptiaca; Citrus x aurantium; Croton macrostachyus; Antidesma venosum; Justicia flava; Phyllanthus muellerianus; Salvia officinalis; Artemisia annua; Maprounea africana*
Dyspepsia	*Artemisia absinthium*
Dystocia	*Bridelia ferruginea*
Edema	*Jatropha curcas; Syzygium guineense; Tetracera poggei; Urtica dioica; Zea mays*
Edema of the lower extremities	*Azanza garckeana*
Elephantiasis	*Crinum ornatum*
Emphysema	*Quassia Africana*
Enuresis	*Caesalpinia decapetala*
Epilepsy	*Annona senegalensis; Azanza garckeana;* Costus lucanusianus; *Elaeis guineensis; Lippia multiflora; Solanum americanum*
Erectile malfunction	*Garcinia huillensis*
Eye troubles	*Maesopsis eminii*
Female infertility	*Ageratum conyzoides; Carica papaya; Elaeis guineensis; Musanga cecropioides; Antidesma venosum; Costus phyllocephalus; Phyllanthus muellerianus; Hymenocardia acida; Tephrosia vogelii; Psidium guajava*
Fever	*Phyllanthus niruri; Alchornea cordifolia; Citrus limon; Citrus x aurantium; Cymbopogon densiflorus; Elaeis guineensis; Gladiolus klattianus;* Isoberlinia tomentosa; *Lantana camara ;Morinda morindoides; *Leucas martinicensis; *Myrianthus arboreus ;Ocimum gratissimum; Persea americana; Physalis angulata; Penianthus longifolius; Tetracera poggei; Mangifera indica; Morinda citrifolia; Momordica charantia*
Filariasis	*Albizia grandibracteata; Tephrosia vogelii*
Fractures	*Ageratum conyzoides; Euphorbia prostrata; Hibiscus esculentus; Indigofera arrecta; Pentaclethra macrophylla; Ocimum gratissimum; Sida acuta*
Frigidity and narrowing of the vagina	*Elaeis guineensis*
Gallbladder disorders	*Artemisia absinthium*
Gallstone	*Vernonia amygdalina*
Gangrene	*Strychnos stuhlmannii*
Gastric hypoacidity	*Artemisia annua; Caesalpinia decapetala*
Gastroenteritis	*Vinca minor*
Gastrointestinal disorders	*Alchornea cordifolia; Ananas comosus; Annona senegalensis; Garcinia huillensis; Pseudolachnostylis maprouneifolia; Piper guineense; Physalis angulata*
Gastric ulcer	*Momordica charantia*
Gastritis	*Cassia occidentalis; Bridelia ferruginea; Brillantaisia patula; Elaeis guineensis;* Isoberlinia tomentosa; *Salvia officinalis; Sida acuta; Antidesma venosum; Jatropha curcas; Citrus limon; Myrianthus arboreus; Vernonia amygdalina; Quassia Africana; Solanum tuberosum; Zanthoxylum chalybeum; Pseudolachnostylis maprouneifolia*
Goiter ringworm	*Jatropha curcas;* Crassocephalum picridifolium
Gonorrhea	*Quassia Africana; Albizia adianthifolia; Bridelia ferruginea; Cassia alata; Citrus x aurantium; Croton macrostachyus;* Costus lucanusianus; *Gladiolus klattianus; Morinda morindoides; Spathodea campanulata; Phyllanthus niruri; Antidesma venosum; Ricinus communis; Jatropha curcas;* Crassocephalum picridifolium*; Maprounea africana; Phyllanthus muellerianus; Strychnos spinosa; Uapaca kirkiana; Pseudolachnostylis maprouneifolia.*
Gout	*Jatropha curcas; Garcinia kola; Phaseolus vulgaris*
Headache	*Ageratum conyzoides; Catharanthus roseus; Elaeis guineensis; Phyllanthus muellerianus; Ocimum gratissimum; Artemisia annua; Mangifera indica; Schwenckia americana; Solanum seretii; Uapaca kirkiana; Vernonia amygdalina; Vernonia shirensis; Vigna sinensis; Vinca minor; Morinda citrifolia*
Helminthiasis	*Phyllanthus niruri; Thomandersia hensii ; Vernonia amygdalina ; Ocimum gratissimum; Quassia africana; Sida acuta; Morinda lucida; Morinda morindoides; Antidesma venosum*
Hemorrhoids	*Annona senegalensis; Bridelia ferruginea; Elaeis guineensis;* Crassocephalum picridifolium*; Nauclea latifolia; Quassia africana; Alchornea cordifolia; Asparagus africanus; Canarium schweinfurthii;* Isoberlinia tomentosa; *Chenopodium ambrosioides; Coleus kilimandschari; Crossopteryx febrifuga; Cyperus alternifolius; Ageratum conyzoides;* Crassocephalum picridifolium*; Ficus exasperata; Hymenocardia acida; Morinda morindoides; Entada abyssinica; Myrianthus arboreus; Ocimum gratissimum; Polygala acicularis; Gladiolus gregarius; Pentaclethra macrophylla; Sida acuta; Phyllanthus muellerianus; Vernonia shirensis; Zingiber officinale; Pterocarpus angolensis; Monodora myristica; Millettia drastica*
Hemorrhage	*Bidens pilosa; Bridelia ferruginea; Citrus limon; Opuntia ficus-indica; Vinca minor*
Hepatitis	*Erythrina abyssinica;* Crassocephalum picridifolium*; Vernonia amygdalina Aloe vera Physalis angulata; Tetracera poggei; Urtica dioica; Mangifera indica*
Hernia	*Aloe congolensis, Annona senegalensis, Antidesma membranaceum; Elaeis guineensis; Erythrina abyssinica; Grewia flava; Harungana madagascariensis; Hymenocardia acida; Morinda lucida; Musa x sapientum; Pentaclethra macrophylla; Phyllanthus niruri;* Leucas martinicensis; *Quassia Africana; Schwenckia americana; Pterocarpus angolensis; Xylopia aethiopica*
Hiccup	*Albizia adianthifolia*
Hip pains	*Zanthoxylum chalybeum*
HIV/Aids	*Panda oleosa*
Hypertension	*Allium cepa; Allium sativum; Catharanthus roseus; Citrus limon;* Isoberlinia tomentosa; Leucas martinicensis; *Pentaclethra macrophylla;* Anacardium occidentale; *Quassia Africana; Harungana madagascariensis; Zea mays*
Hypotension	*Allium sativum; Acacia polyacantha;* Psorospermum corymbiferum
Indigestion	*Albizia adianthifolia, Anana comesus; Cassia occidentalis*
Infected wounds	*Ochna schweinfurthiana*
Infections	*Adenia gummifera; Allium sativum; Antidesma venosum; Arachis hypogaea; Cymbopogon densiflorus; Gongronema latifolium; Morinda lucida; Nauclea latifolia; Rauvolfia caffra; Vernonia amygdalina; Zingiber officinale; Moringa oleifera*
Infertility	*Uapaca kirkiana; Millettia drastica*; *Musa x sapientum; Zanthoxylum chalybeum, Pseudolachnostylis maprouneifolia*
Inflammation	*Ageratum conyzoides; Raphia gentiliana; Physalis angulata; Physalis peruviana*
Influenza	*Chenopodium ambrosioides; Ocimum gratissimum*
Insomnia	*Catharanthus roseus*
Intercostal (or chest) pain	*Elaeis guineensis*
Interruption of the menstruation without being pregnant	*Bridelia ferruginea*
Intestinal worms	*Allium sativum; Antidesma venosum; Bridelia ferruginea; Carica papaya; Catharanthus roseus; Chenopodium ambrosioides; Entada abyssinica; Garcinia huillensis; Garcinia kola; Ipomoea spathulata; Jatropha curcas; Morinda morindoides; Penianthus longifolius; Strychnos spinosa; Syzygium guineense; Tephrosia vogelii; Vernonia shirensis; Zingiber officinale; Musa x sapientum; Millettia drastica; Maprounea africana*
Irritable bowel	*Carica papaya*
Jaundice	*Acacia karroo; Carica papaya; Eminia polyadenia; Harungana madagascariensis; Jatropha curcas; Musanga cecropioides; Rhynchosia insignis; Thomandersia hensii; Terminalia mollis.*
Joint pain	*Aloe congolensis, Annona senegalensis; Costus phyllocephalus; Morinda morindoides; Lippia multiflora*
Kidney stone	*Phaseolus vulgaris; Zea mays*
Laryngitis	*Bridelia ferruginea*
Leishmaniasis	*Morinda lucida*
Lice	*Rauvolfia vomitoria*
Lumbago	*Elaeis guineensis*
Madness	*Elaeis guineensis; Polygala acicularis*
Malaria	*Crossopteryx febrifuga; Alchornea cordifolia; Acacia polyacantha; Albizia adianthifolia; Antidesma venosum; Artemisia annua; Azadirachta indica; Cassia occidentalis; Cymbopogon citratus; Catharanthus roseus; Jatropha curcas; Lantana camara; Morinda lucida; Morinda morindoides; Citrus x aurantium; Coleus kilimandschari; Cymbopogon densiflorus; Eucalyptus globulus; Garcinia kola; Indigofera arrecta; Musanga cecropioides; Myrianthus arboreus; Ocimum gratissimum;* Parinari capensis*; Pentaclethra macrophylla; Phyllanthus niruri; Rauvolfia caffra; Thomandersia hensii; Vernonia amygdalina; Harungana madagascariensis; Momordica charantia; Penianthus longifolius; Vernonia shirensis; Rauvolfia vomitoria; Piliostigma thonningii; Piper guineense; Physalis angulata; Physalis peruviana; Moringa oleifera; Monodora myristica; Eucalyptus globulus; Quassia africana*
Male impotence	*Elaeis guineensis; Phyllanthus muellerianus; Cassia petersiana; Citrus limon; Sida acuta; Balanites aegyptiaca; Cola nitida; Kigelia africana; Penianthus longifolius; Schwenckia americana; Lippia multiflora; Xylopia aethiopica*
Mastitis	*Aloe congolensis; Ocimum gratissimum, Pterocarpus angolensis*
Measles	*Aristolochia hockii; Cymbopogon citratus; Costus phyllocephalus; Thomandersia hensii*
Migraine	*Elaeis guineensis; Ocimum gratissimum; Vinca minor*
Mycosis	*Cassia alata; Stachytarpheta indica*
Nephritis	*Zea mays*
Neuralgia	*Musa x sapientum*
Oligospermia	*Phyllanthus muellerianus*
Oliguria	*Maprounea africana*
Oral cavity	*Euphorbia prostrata*
Oropharyngeal diseases	*Salvia officinalis; Lantana camara*
Otitis	*Citrus x aurantium; Ocimum gratissimum;* Crassocephalum picridifolium*; Zanthoxylum chalybeum; Tephrosia vogelii*
Oxytocic	*Sida acuta*
Pain	*Quassia Africana; Phyllanthus niruri; Persea americana*
Paralysis	*Brassica juncea; Olax obtusifolia*
Pneumonia	*Acacia polyacantha; Elaeis guineensis;* Psorospermum corymbiferum*; Pseudolachnostylis maprouneifolia*
Poisoning antidote	Isoberlinia tomentosa; *Gongronema latifolium; Vernonia amygdalina; Pseudolachnostylis maprouneifolia*
Poliomyelitis	*Xylopia aethiopica*
Premature ejaculation	*Elaeis guineensis*
Psychosomatic disorders	*Solanum americanum*
Premature delivery	*Cymbopogon citratus*
Prevention of tetanus	*Pseudolachnostylis maprouneifolia*
Pruritus	*Jatropha curcas*
Rashes with itching	*Abrus precatorius; Solanum americanum; Vernonia amygdalina*
Rheumatism	*Allium cepa; Bridelia ferruginea; Dalbergia boehmii; Elaeis guineensis; Quassia africana; Morinda morindoides; Costus phyllocephalus; Erythrophleum africanum; Garcinia huillensis; Ocimum gratissimum; Pentaclethra macrophylla; Harungana madagascariensis; Urtica dioica; Lantana camara; Xylopia aethiopica; Schwenckia americana.*
Scabies	*Elaeis guineensis; Vernonia amygdalina; Quassia africana; Jatropha curcas*
Schistosomiasis	*Annona senegalensis; Balanites aegyptiaca; Citrus limon; Cymbopogon densifloru; Eminia polyadenia; Entada abyssinica; Garcinia huillensis; Harungana madagascariensis; Hymenocardia acida; Strychnos innocua; Strychnos spinosa; Syzygium guineense; Terminalia mollis; Vernonia shirensis; Piliostigma thonningii; Pterocarpus angolensis; Ochna schweinfurthiana; Maytenus senegalensis; Maprounea africana*
Sciatic neuralgia	*Elaeis guineensis; Ocimum gratissimum; Schwenckia americana*
Sickle cell disease	*Adansonia digitate*; *Annona senegalensis; Bridelia ferruginea; Carica papaya; Coleus kilimandschari; Combretum celastroides; Costus phyllocephalus; Cymbopogon densiflorus; Jatropha curcas; Terminalia ivorensis; Mitragyna stipulosa; Persea americana; Thomandersia hensii; Bougainvillea spectabilis; Morinda lucida; Hymenocardia acida; Harungana madagascariensis; Vigna sinensis; Maesopsis eminii.*
Sinusitis	*Erythrina abyssinica*
Skin infections	*Albizia grandibracteata; Allium cepa; Brassica oleracea*
Skin rash	*Acacia polyacantha; Tephrosia vogelii*
Smallpox	*Morinda morindoides*
Snakebites	*Thomandersia hensii ; Euphorbia prostrata; Rauvolfia caffra*
Sore throat	*Aframomum melegueta; Citrus limon; Euphorbia prostrata; Ficus exasperata; Piper guineense*
Spasms	*Acacia polyacantha;* Psorospermum corymbiferum
Splenomegaly	*Aloe congolensis; Annona senegalensis; Elaeis guineensis; Tithonia diversifolia*
Sprain	*Hibiscus esculentus*
Stomach pain	*Antidesma venosum; Basella alba; Crossopteryx febrifuga; Physalis angulata; Lantana camara; Phyllanthus niruri; Citrus limon; Quassia africana; Jatropha curcas; Phyllanthus muellerianus; Ageratum conyzoides;* Crassocephalum picridifolium*; Solanum americanum*
Sweating	*Salvia officinalis*
Swollen breasts	*Morinda lucida*
Swollen gums	*Ricinus communis*
Swollen testicles	*Pseudolachnostylis maprouneifolia; Ricinus communis*
Syphilis	*Albizia adianthifolia; Antidesma venosum; Aristolochia hockii; Asparagus africanus;* Isoberlinia tomentosa; *Pseudolachnostylis maprouneifolia; Ricinus communis; Strychnos innocua; Strychnos spinose; Strychnos stuhlmannii; Terminalia mollis; Lonchocarpus katangensis; Maprounea africana*
Tachycardia	*Musanga cecropioides; Thomandersia hensii*
Testicular disappearance	*Annona senegalensis; Elaeis guineensis*
Tiredness	*Costus phyllocephalus*
Tooth decay	*Ageratum conyzoides; Antidesma venosum; Dalbergia boehmii; Elaeis guineensis; Lonchocarpus katangensis; Myrianthus arboreus; Phyllanthus muellerianus; Ricinus communis; Swartzia madagascariensis;* Psorospermum corymbiferum*;Uapaca kirkiana;* Anacardium occidentale; *Pseudolachnostylis maprouneifolia; Mangifera indica; Millettia drastica; Maprounea africana; Acacia polyacantha*
Trypanosomiasis	*Annona senegalensis; Morinda lucida*
Tuberculosis	*Abrus precatorius; Azadirachta indica; Bridelia ferruginea; Canarium schweinfurthii; Citrus limon; Eucalyptus globulus; Hymenocardia acida; Myrianthus arboreus; Ocimum gratissimum; Chenopodium ambrosioides; Costus phyllocephalus; Garcinia huillensis; Schwenckia americana; Rauvolfia caffra; Vernonia amygdalina; Vitex madiensis; Rauvolfia vomitoria; Momordica charantia; Lippia multiflora*
Typhoid fever	*Antidesma venosum; Arachis hypogaea; Morinda morindoides*
Urinary infections	*Albizia grandibracteata; Alchornea cordifolia; Bidens pilosa; Eminia polyadenia; Maesopsis eminii; Mangifera indica; Spathodea campanulata; Strychnos cocculoides; Vitex madiensis.*
Uterine bleeding	*Urtica dioica*
Uterine contraction	*Uapaca kirkiana*
Vaginal infections	*Acacia karroo*; *Kigelia Africana; Acacia polyacantha; Salvia officinalis*
Venereal diseases	*Crotalaria spinosa*
Verminous	*Cassia sieberiana*
Vitiligo	*Elaeis guineensis*
Vomitings	*Basella alba; Cassia occidentalis; Piper guineense; Vinca minor*
Weak immunity system	*Allium sativum*
Whitlow	*Elaeis guineensis*
Wounds	*Morinda morindoides; Annona senegalensis; Bidens pilosa; Quassia africana; Jatropha curcas*
Yellow fever	*Elaeis guineensis; Harungana madagascariensis*

### 4.2 Analysis of Pharmacological Data

#### 4.2.1 Preclinical Studies

**FIGURE 6:**
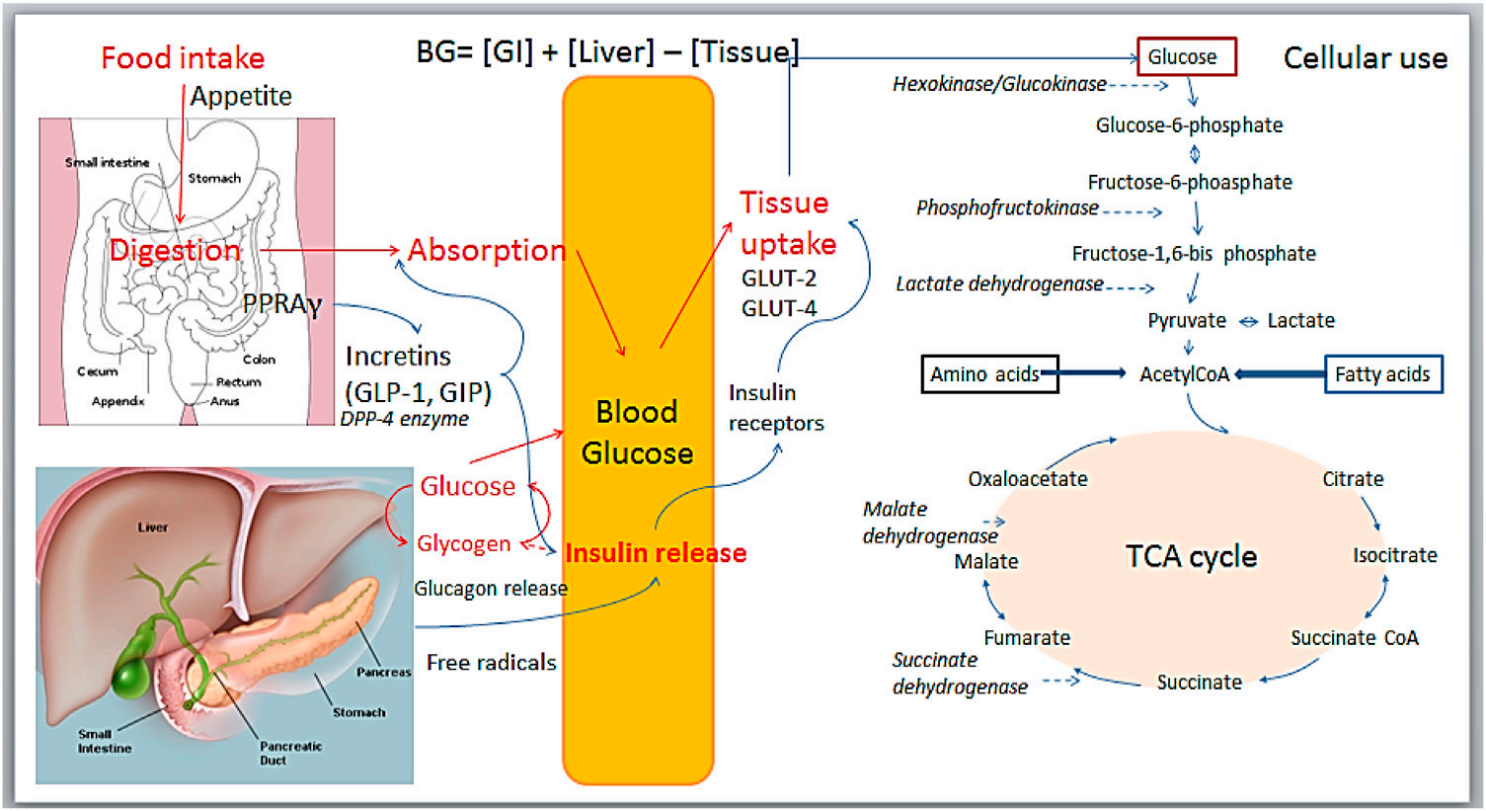
Illustrative sites and pathways of antidiabetic bioactivity.

Different strategies and pathways are used to determine the mechanism of antidiabetic agents, as shown in Figure 6. No study explored in-depth pharmacological mechanisms of action, but all speculated over different modulating metabolic pathways, including 1) Reducing food intake; 2) Reducing carbohydrate digestion and absorption (alpha-amylase, alpha-glucosidase inhibition); 3) Increasing glycogenesis or reducing glycogenolysis and cholesterol synthesis; 4) Free radical scavenging action; 5) Insulin release and pancreas β-cells regeneration; 6) Enhancing glucose transport GLUT4 translocation; 7) dipeptidyl peptidase-4 (DPP-4) inhibition; 8) (PPARs); 9) Insulin-mimetic activity; 10) Modulation on Krebs cycle enzymes.

The analysis of the accurate data for all 213 plants listed showed that most studies used rats and mice, and in a few cases, guinea pigs and rabbits. Both streptozocin (35.55%) and alloxan (24.64%) represented 60.19% of all *in vivo* reported study models. Streptozotocin presents many advantages over alloxan, including its longer half-life, more productive, stable, and selective to islet beta cells, less toxic, and causing less mortality in animal models (Lenzen, 2008; Wang-fischer and Garyantes, 2018). The majority of the bioactivity investigations link the antioxidant or free radical-scavenging activity with the pathophysiology of Diabetes. However, currently, the *in vitro* antioxidant model using, for example, DPPH and the others, is not pharmacologically relevant. It can be used as a chemical screening tool. Only *in vivo* or cell-based models remain relevant (Heinrich et al., 2020). Enzymes are a frequent pharmacological target for establishing the mechanism of action of new drugs. Upon *in vitro* studies, alpha-glucosidase activity inhibition was the most common investigation (45.3%), followed by inhibition of PTP1B (13.8%), alpha-amylase (9.7%), DPP-4 (1.4%), and 11ß-HSD1 activity (1.0%). Additionally, in cell lines studies, glucose uptake (28.0%) was to be the most commonly used, followed by glucose uptake regulation markers such as GLUT4 translocation and expression levels (9.7%) and PPAR (9.6%) (Munhoz and Fröde, 2017). Some examples of studies are given below.

*Azadirachta indica* aqueous leaf extract (400 mg/kg bw) improved levels of BG, serum insulin, lipid profile, insulin signaling molecules, and GLUT4 proteins in the tissue of high-fat fructose-induced type-2 diabetic male rat after 30 days of treatment, compared to the control group. In Goto-Kakizaki rats, the acetone extract of *Syzygium cumini* seed was a potent inhibitor of alpha-glucosidase hydrolysis of maltose compared to untreated control animals (Shinde et al., 2008). Moreover, hepatic tissue demonstrated increased PPARɣ and PPARα protein expressions (Sharma et al., 2012). *Oryza sativa* extracts significantly elevated glucose uptake, GLUT1, and GLUT4, mRNA levels (Boue et al., 2016). Ethanol extract induced a significant gain in GLUT4 on plasma membranes of L6-GLUT4myc muscle cells at no cytotoxic concentrations (Kadan et al., 2013). Choosing an experimental model is not easy and usually depends on many factors. Ideally, the experiments should be carried out in several different models, considering that none of them ultimately reflects the complexity of human diabetes mellitus type DMT2 and that precautions should be taken to extrapolate the findings to the clinical practice (Arias-Díaz and Balibrea, 2007).

*A. adianthifolia* is also used to treat syphilis, hiccups, diarrhea, malaria, indigestion, blueness, and an aphrodisiac. Oral administration of 500 mg/kg of plant extract reduced hyperglycemia by 57% in guinea pigs subject to OGTT (Amuri et al., 2017). *Albizia grandibracteata* is used in filariasis and skin and urinary tract infections. *A. garckeana* is used in epilepsy and edema of the lower limbs. Oral administration of 500 mg/kg bw aqueous extract under OGTT conditions reduced fasting blood sugar to 36.9% compared to 49.6% of glibenclamide as the reference medicine (Amuri et al., 2017). Certain parts of the plant may be toxic or contain cytotoxic compounds, particularly with gossypol for non-ruminant animals (Randel et al., 1992). *Gladiolus gregarius* is used to treat hemorrhoids and back pain. *Gladiolus klattianus* is used for gonorrhea and fever. Under OGTT conditions, the aqueous extract of *G. klattianus* reduced 35% of blood sugar after 60 min (Amuri et al., 2017). *Panda oleosa* Pierre has been proposed for HIV/AIDS. The aqueous extract of *P. oleosa* (25–100 mg/kg) significantly reduced glucose levels in a dose-dependent manner in rabbits under OGTT conditions (Muhoya et al., 2017). *Vitex madiensis* is used in asthma, anemia, diarrhea, tuberculosis, cough, urinary tract infections, and intestinal amebiasis. The aqueous extract of *V. madiensis* (500 mg/kg bw) reduced hypoglycemia to 43% compared to 55% obtained with glibenclamide (Amuri et al., 2017). *Raffia gentiliana* is used for inflammation. Oral administration of aqueous fruit extracts in mice under OGTT conditions demonstrated 27 and 56% reduction after one and 2 hours (Mpiana et al., 2013).

Of the 213 plant species listed, 134(62.91%) underwent experimental studies in animals or *in vitro*, while only 8.92% reached the clinical trial phase. Inside DRC, only seven plants shown in Table 4 have been studied in animals. A critical analysis of the seven studies carried out inside DRC showed low quality (grade = 4–5). The majority (85.71%) used a single dose in antidiabetic evaluation. However, the *Panda oleosa* study used three-dose rages (25, 50, and 100 mg/kg body weight). Overall, it is not easy to define an exact upper cut-off dose. In most cases, an oral dose range of 100–200 mg/kg body weight for plant extracts *in vivo* investigations should be considered the upper limit (Heinrich et al., 2020). Experiments on *Albizia adianthifolia, Azanza garckeana, Gladiolus klattianus*, and *Rauvolfia caffra* extracts used the highest dose (500 mg/kg bw) calculated from the human patients of 60 kg treated with 750 ml of plant extract (corresponding to 250 g of dried herbal material per day). Par the way, differences in doses that normalize interspecies variation should be taken into account (Nair and Jacob, 2016).

**TABLE 4:** Local plants studied for antidiabetic effect in animals.

Plant	Ecology	Form	Part	Animal	Model	Dose range	Quality score	References
*Albizia adianthifolia*	T, Fo, At	D	Stem bark	Guinea pig	OGTT	500 mg/kg^a^	5/10 low	Amuri et al. (2017)
*Azanza garckeana*	Tp/Sh, Cult, SA.	D	Leaf	Guinea pig	OGTT	500 mg/kg^a^	5/10 low	Amuri et al. (2017)
*Gladiolus klattianus*	T, Cult, Sarc	M	Bulb	Guinea pig	OGTT	500 mg/kg^a^	5/10 low	Amuri et al. (2017)
*Panda oleosa*	Anh, Cult, Cosm	D	Bark	Rabbit	OGTT	25, 50 and 100 mg/kg	5/10 low	Muhoya et al. (2017)
*Raphia gentiliana*	Sh, Cult, At	M	Fruit	Mouse	OGTT	200 mg/kg	5/10 low	Mpiana et al. (2013)
*Rauvolfia caffra*	T, Fo, Pan	D	Root	Guinea pig	OGTT	500 mg/kg^a^	5/10 low	Amuri et al. (2017)
*Vitex madiensis*	T,GSZ,Sav	D	Leaves	Guinea pig	OGTT	500 mg/kg^a^	5/10 low	Amuri et al. (2017)

T(tree); Fo(forest); At(Afro-tropical); Tp(perennial-tree); Sh (Shrub), Cult(Cultivar); SA(South Africa); Sarc(Sarcochores); Anh(annual herb); Cosm(cosmopolitan); Pan(pantropical); Sav(savannah); GSZ(Guinean and Zambian); D(Decoction), M(Maceration); OGTT (oral glucose tolerance test).

a Justification of dose.

Temperature and humidity were not reported. The effeteness of the treatment was based on the capacity of the extract to reduce baseline glycemia (hypoglycemia effect) or the capacity to reduce induced hyperglycemia; this varied between 25 and 75%, compared to reference drugs (glibenclamide and metformin). According to (Baker et al., 2014), over 85% of published animal studies do not describe randomization or blinding, and over 95% lack the estimation of sufficient sample size needed for detecting actual effects.

#### 4.2.2 Toxicological Data

**FIGURE 7:**
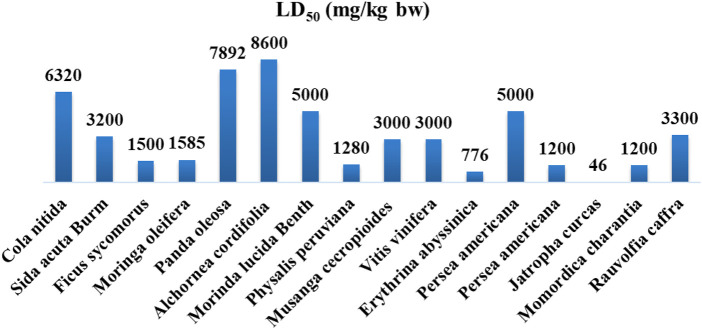
Comparative range of LD_50_ for some plants.

For acute toxicity, Figure 7 shows comparative values of LD_50_ reported for *Cola nitida, Sida acuta, Ficus sycomorus*, *Moringa oleifera, Panda oleosa, Alchornea cordifolia, Morinda lucida, Physalis peruviana, Musanga cecropioides, Vitis vinifera, Erythrina abyssinica, Persea americana, Jatropha curcas, Momordica charantia,* and *Rauwolfia caffra*. Almost all plants but *Jatropha curcas* are relatively non-toxic (LD_50_ > 500 mg/kg).

The highest value of LD_50_ was observed at 8,600 mg/kg bw in rodents with an oral administration of *Alchornea cordifolia.* The bark extract of Panda oleosa was practically non-toxic in guinea pigs with an LD_50_ of approximately 7,892 mg/kg bw; no signs of intoxication were observed with oral doses less than 2,000 mg/kg. However, at doses above 6,000 mg/kg, poor mobility, poor appetite, anuria, and death have been noted in animals (Katemo et al., 2018). The administration of the aqueous extract from the bark of *Ficus benghalensis* suggested an LD_50_ > 5,000 mg/kg. In some cases, the toxic effects depended on sex, like *Alchornea cordifolia*, which showed different values of LD_50_ in mice male compared to female animals (8,600 mg/kg in male and 3,800 mg/kg female) (Djimeli et al., 2017). Despite low acute toxicity, many plants exhibit some significant sub-chronic toxicity. *Caesalpinia bonduc* extract showed hematological changes after a subchronic study for 28 days at a dose up to 400 mg/kg bw in rats (Ogunlana et al., 2013). Except for the ripe fruit, solanine and other alkaloids present in all parts of Solanum americanum are toxic (Kuete, 2014). Aloe-emodin (anthraquinone) from *Aloe species* could be mutagenic or/and genotoxic in organs (Lynch et al., 2011). A daily and prolonged administration (28 days) of resveratrol in *Vitis vinifera* exhibited nephrotoxicity in the rat at the high dose (3000 mg/kg bw). Extracts of *Aframomum melegueta* (286–345 mg/kg bw) and *Artemisia annua* (300 mg/kg bw) produced a toxic effect on the development of fetus by discontinuation of first trimester pregnancies in rats (Inegbenebor et al., 2009; Abolaji et al., 2012). Leaf methanol extract of *Jatropha curcas* decreased the number of live fetuses and increased placental weight (Teixeira et al., 2017). Bulbs’ aqueous extract of *Crinum ornatum* had caused significant effects (Central Nervous System), including losing appetite, slow movement, depression, less aggression, and lying at the corners of the cage (Lawal and Dangoggo, 2014). *Erythrina abyssinica* showed similar nervous effects in mice, significantly decreasing motility, sedation, frequent urination, and tremors during the first 6 h after drug administration at different doses (Bunalema et al., 2011). Some compounds in *Salvia officinalis* (Camphor, thujone, and terpene ketones) are considered the most toxic. Their consumption is not recommended in pregnancy and lactation because they are harmful to the fetus and newborn (Ghorbani and Esmaeilizadeh, 2017). A methanol extract (500 and 1,000 mg/kg/day) of *Catharanthus roseus* in the subacute investigation for 14 days showed inevitable mortality and presented some of the signs of intoxication on the study of the liver and kidney rats (Kevin et al., 2012). Sometimes, there are some contradictions in findings from different authors on toxicological studies in animals. In *Cassia occidentalis*, Lagarto et al. (2011) and Mishra et al. (2018) are contradictory. The first group did not report any toxicological signs in biochemical, hematological, and morphological markers, while the second group noticed some changes.

#### 4.2.3 Clinical Trials

Data from the present study showed the lack of local clinical trials of antidiabetic plants used to manage Diabetes in the DRC. Of seven native herbals, only *Raphia gentiliana* fruit extract was given to 25 males and 20 females, aged 18–50 years old, with normal blood sugar levels (Mpiana et al., 2013). The approach followed by the authors did not comply with any clinical trial requirements, and instead, their behavior went like traditional healers themselves.

Table 5 illustrates the assessment of the quality of clinical trials of antidiabetic plants using the Jadad scale for reporting randomized controlled trials based on randomization, blinding, withdrawals, and dropout methods.

**TABLE 5:** The interpretation of Jadad score on Clinical trials of antidiabetic plants found in DRC.

*Plant used*	Used part/Preparation	Author and year	Randomization	Blinding	Withdrawals and dropouts	Total
*Allium cepa*	Fresh pods	Jafarpour-Sadegh et al. (2017)	2	2	1	5
*Allium sativum*	Pods	Ashraf et al. (2011)	1	2	0	3
*Aloe vera*	High molecular weight fractions	Yagi et al. (2009)	0	0	0	0
*Balanites* aegyptiaca	Fruits	Rashad et al. (2017)	2	2	1	5
*Carica papaya*	Fermented papaya	Raffaelli et al. (2015)	0	0	0	0
*Elaeis guineensis*	Standardized leaf extract	Kalman et al. (2013)	1	2	0	3
*Laurus nobilis*	Ground leaves	Khan et al. (2009)	1	0	0	1
*Momordica charantia*	Fruit powder	Kim et al. (2020)	1	1	1	3
*Morinda cordifolia*	Juice from fermented fruit puree	Algenstaedt et al. (2018)	0	0	0	0
*Moringa oleifera*	Leaf powder	Leone et al. (2018)	0	2	0	2
*Rauvolfia-Citrus*	Leaf powder	Campbell-Tofte et al. (2011)	1	2	1	4
*Raphia gentiliana*	Fruits	Mpiana et al. (2013) ^a^	0	0	0	0
*Salvia officinalis*	Leave powder	Kianbakht and Dabaghian, (2013)	1	2	1	4
*Terminalia chebula*	Fruit aqueous extract	Pingali et al. (2020)	2	2	1	5
*Trigonella foenum-graecum*	Seed powder	Hadi et al. (2020)	2	1	1	4
*Urtica dioica*	Leaf extract	Kianbakht et al. (2013)	2	2	1	5
*Vernonia amygdalina*	Leaf juice	Okolie et al. (2008)	2	0	0	2
*Zea mays*	Maize starch	Sands et al. (2009)	1	0	0	1
*Zingiber officinale*	Rhizome powder	Shidfar et al. (2015)	1	2	0	3

a Only study carried out in DRC; Score ≥ 3(Good quality); Score < 3 (Poor quality).

In general, out of 213 plants censored, approximately 8.92% (*n* = 19) have been validated by clinical evidence. These are *Allium cepa* (Jafarpour-Sadegh et al., 2017), *Allium sativum* (Ashraf et al., 2011), *Balanites aegyptiaca* (Rashad et al., 2017), *Citrus aurantium* (Campbell-Tofte et al., 2011), *Elaeis guineensis* (Kalman et al., 2013), *Laurus nobilis* (Khan et al., 2009), *Momordica charantia* (Kim et al., 2020), *Morinda cordifolia* (Algenstaedt et al., 2018), *Moringa oleifera* (Leone et al., 2018), *Rauvolfia-Citrus* (Campbell-Tofte et al., 2011), *Salvia officinalis* (Kianbakht and Dabaghian, 2013), *Terminalia chebula* (Pingali et al., 2020), *Trigonella foenum-graecum* (Hadi et al., 2020); *Urtica dioica* (Kianbakht et al., 2013), *Vernonia amygdalina* (Okolie et al., 2008), *Zea mays* (Sands et al., 2009), and *Zingiber officinale* (Shidfar et al., 2015).

For example, a double-blind, placebo-controlled, randomized clinical trial conducted on 20–60 year-old DMT2 patients who did not receive insulin showed that 3 months supplementation of 3 g of ginger (*Zingiber officinale*) improved glycemic indices, total antioxidant capacity, malondialdehyde, C-reactive protein, serum paraoxonase, dietary intake, and physical activity, measured at the beginning and end of the study, and after 12 h fasting compared to control groups. A randomized, placebo-controlled, parallel-group study with 42 treated patients treated with leaf hydroethanolic extract (500 mg/8 h for 3 months) and 44 as placebo groups showed that the *Salvia officinalis* leaves lowered fasting glucose and HbA1c the baseline at the endpoint with no adverse effects reported. A clinical trial on a juice extract from the fruit of *Morinda cordifolia* (2 ml/kg bw once a day) in patients with DMT2, after 90 days of treatment, presented a significant reduction of morning BG in several cases, an improvement of hyperglycemia status. In a prospective, randomized, double-blind, placebo-controlled clinical investigation, the administration of *Terminalia chebula* (250 and 500 mg/kg bw, for 12 weeks) in 60 diabetic patients significantly improved the endothelial function (reflection index) compared to placebo (−2.55 ± 1.82%, and −5.21 ± 2.41%, respectively). In an 8-weeks randomized controlled clinical trial study of the effect of *Trigonella foenum-graecum* intake seed in 50 patients with T2DM, the plant significantly reduced fasting blood glucose. It improved some liver and kidney function compared with control interventions.

A randomized, double-blind, placebo-controlled clinical trial of *Urtica dioica* leaf extract (500 mg/8 h, 3 months) combined with conventional oral antihyperglycemic drugs was conducted in 46 treated patients vs 46 placebo groups. At the endpoint, the extract significantly lowered the blood levels of fasting glucose, 2 h postprandial glucose, and HbA1c, without significant effects on other hepatic or cardiovascular parameters, vs the placebo. All considered these results demonstrated that nettle is safe and may have a beneficial effect on glycemic control in patients with advanced DMT2 needing insulin therapy. *Vernonia amygdalina* elicited a significant reduction in BG levels at the most postprandial time points and area-under-curve.

Unfortunately, many studies were carried out in poor quality conditions, with unclear randomization methods, threats to blinding, and lack of baseline demographics (Rios et al., 2015). The interpretation of Jadad score on clinical trials reviewed showed that studies conducted on *A. cepa, A. sativum, B.* ae*gy*ptiaca*, E. guineensis, M. charantia, R. vomitoria, S. officinalis, U. dioica,* and *Z. officinale* presented excellent quality (Jadad score ≥ 3)(Hartling et al., 2011). In addition to the effectiveness of the plant materials (extracts, isolated compounds), clinical trials must include other vital parameters to an antidiabetic evaluation in particular glycosylated hemoglobin A1c (HbA1c), personal medication, insulin, glycogen, lipid and protein profiles, and severity of adverse effects, patient’s risk factors, ease of use, patient’s financial situation, etc. (Chaudhury et al., 2017).

### 4.3 Analysis of Phytochemical Data

**FIGURE 8:**
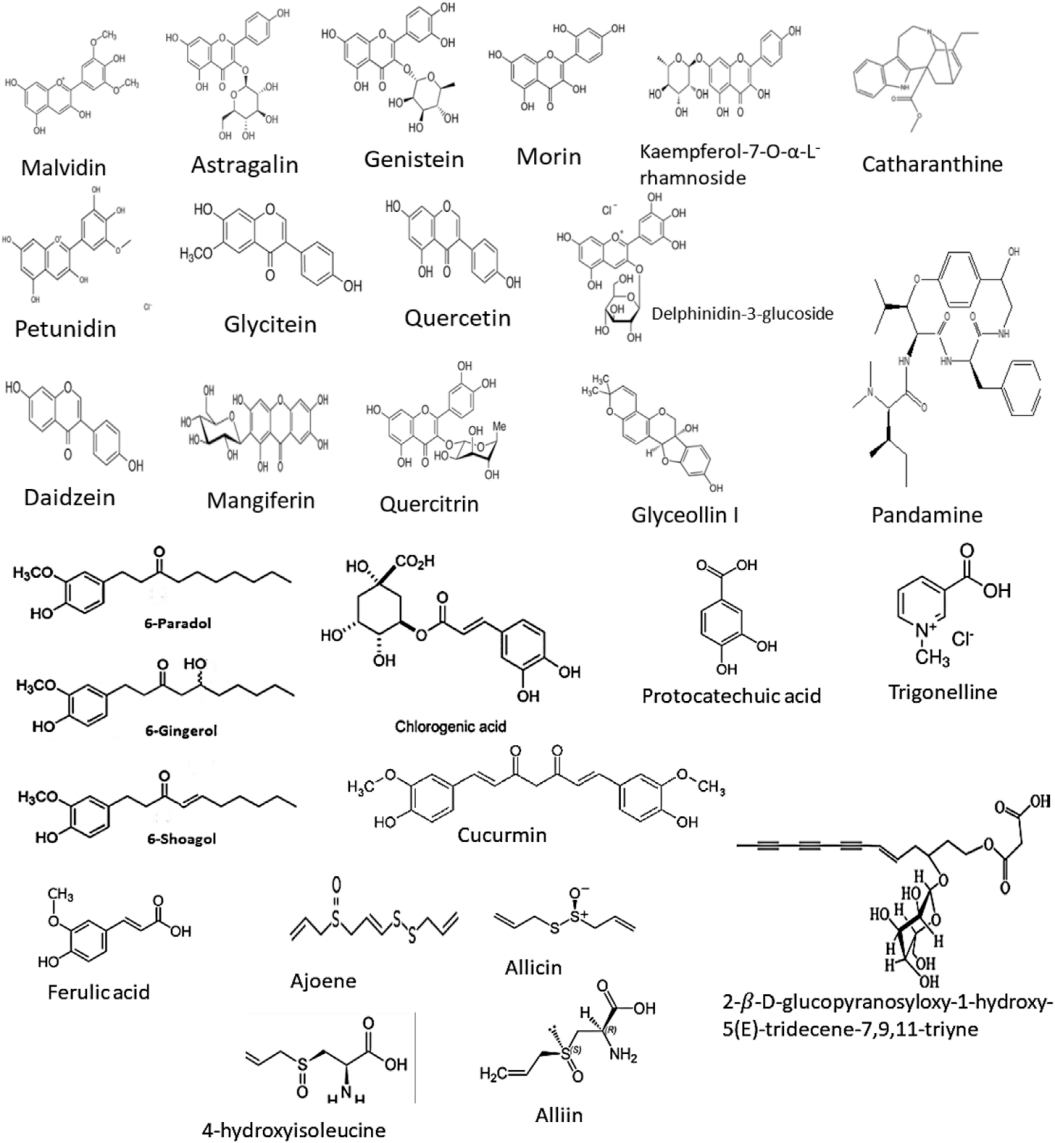
Some bioactive components isolated.

Various second metabolites have been identified and isolated, as shown in Table 6 and Figure 8. Qualitative and quantitative content may vary with the soil where the plants are growing.

**TABLE 6:** Major phytochemicals of each plant with demonstrated antidiabetic activity.

*Scientific Names*	Antidiabetic compounds
*Abrus precatorius*	Luteolin, lupenone, 24-methylnecycloartenone, and luteolin Vadivel et al. (2011); Yonemoto et al. (2014)
*Aframomum melegueta*	Arylalkanes, 6-paradol, 6-shogaol, 6-gingerol, 6-gingeredione, a pentacyclic triterpene, oleanolic acid isolated from the fruit Sugita et al. (2013); Mohammed et al. (2017).
*Ageratum conyzoides*	Precocene II Adebayo et al. (2010), and Kaempferol Tahora et al. (2018),
*Allium cepa*	Ferulic acid, alliin Tang et al. (2008), agavasaponin C Tang et al. (2008), flavonoid alliuocide G Mohamed (2008), quercetin, sulfur compounds, alcohols, aldehydes, esters, and other chemical groups. S-methyl cystein sulfoxide, S-allyl cysteine and diallyl thiosulfanate Kim et al. (2010), Bakhshaeshi et al. (2012), Noor et al. (2013), Cepadial D ; 1,3,11a-trihydroxy-9-(3,5,7-trihydroxy-4H-1-benzopyran-4-on-2-yl)-5a-[4-(β-D-glucopyranosyloxy)-3-hydroxyphenyl]-5,6,11-hexahydro-5,6,11- trioxanaphthacene-12-one ; and 1,3,11a-trihydroxy-9-(3,5,7-trihydroxy-4H-1-benzopyran-4-on-2-yl)-5a-[1,3,11a-trihydroxy-5a-(3,4-dihydroxyphenyl)-5,6,11-hexahydro-5,6,11-trioxanaphthacene-12- on-9-yl]-5,6,11-hexahydro-5,6,11-trioxanaphthacene-12-on Vu et al. (2020).
*Allium sativum*	S-allylcysteine sulfoxide, alliin, diallyl trisulfide Liu et al. (2007); Mikaili et al. (2013), isoeruboside B, agavasaponin C, proto-iso-erubisoide B, 2-Vinyl-4H-1,3-dithiin Tang et al. (2008), allicin, diallyl disulfide, diallyl sulfide, ajoene, and allyl mercaptan Bayan et al. (2014).
*Aloe vera*	Lophenol, 24-methyl-lophenol, 24-ethyl-lophenol, cycloartanol and cycloartanol Misawa et al. (2012), aloeresin A Chang et al. (2013b), aloerisin Jong-anurakkun et al. (2008), aloe-emodin-8-O-glucoside, polysaccharides Salehi et al. (2018), aloin, barbaloin, isobarbaloine, aloetic acid, emodin, cinnamic acid, crysophanic acidleucine, isoleucine, alanin, glucomannan, cellulose, mannose, zinc, glucosamines Bharti et al. (2018).
*Anacardium occidentale*	Anacardic acid Tedong et al. (2010), lectin MacIel et al. (2012)
*Arachis hypogaea*	Leucocyanidin, stigmasterol Tang et al. (2008), resveratrol Gothai et al. (2016) phenolic compounds such as catechin, caffeic acid, epicatechin, p-coumaric acid, rutin, trans-ferulic acid, isoquercitri, resveratrol, luteolin, quercetin, trans-cinnamic acid, chrysoeriol Park et al. (2017).
*Artemisia absinthium*	α-and ß-thujones Daradka et al. (2014)
*Azadirachta indica*	3-Deacetyl-3-cinnamoyl-azadirachtin Jalil et al. (2013), 4'-methyl-quercetin-7-*O*-β-D-glucuronopyranoside, 2,3-hexahydroxydiphenoyl-(α/β)-D-(4)C1-glucopyranose, avicularin, castalagin, quercetin-3-O-glucoside Abdelhady et al. (2016) sistosterol, stigmasterol, campestrol, squalene, nimbiol and others Sanni et al. (2019).
*Balanites aegyptiaca*	Furostanol saponins Ezzat et al. (2017), balanitin 1 and 2, diosgenin, stigmast-4-en-3-ol, pure saponins Hassanina et al. (2018)
*Bidens pilosa*	Cytopiloyne, 2- β -D-Glucopyranosyloxy-1-hydroxytrideca-5,7,9,11-tetrayne Chang et al. (2013a), polyynes Bartolome et al. (2013), 3-β-D-glucopyranosyl-1-hydroxy-6(*E*)-tetradecene-8,10,12-triyne; 2-β-D-glucopyranosyloxy-1-hydroxy-5(*E*)-tridecene-7,9,11-triyne Chang et al. (2013b).
*Bougainvillea spectabilis*	Pinitol, βsitosterol, quercetin, quercetin 3-O-α-L-rhamnopyranoside Jawlal et al. (2013).
*Brassica juncea*	Cinnamic acid Guzman (2014), Kaempferol Gothai et al. (2016), aniline Sundowo et al. (2018)
*Brassica oleracea*	Cinnamic acid Guzman (2014), kaempferol Gothai et al. (2016)
*Bridelia ferruginea*	Epigallocatechin, epigallocatechin gallate Bakoma et al. (2018)
*Caesalpinia decapetala*	Apigenin-7-rhamnoside, astragalin, 6-hydroxy kaempferol, quercitrin Parveen et al. (2017)
*Calendula officinalis*	Caffeic acid, aesculetin, quercetin and isorhamnetin Olennikov and Kashchenko (2014).
*Carica papaya*	Flavonoids, alkaloids, saponin, and tannin Chang et al. (2013b)
*Cassia alata*	Emodin Uwazie et al. (2020)
*Cassia occidentalis*	Flavonoids Gupta et al. (2017)
*Catharanthus roseus*	Gallic acid, chlorogenic acid, flavonoids Rianika and Robert (2007), vindoline I, vindolidine II, vindolicine III and vindolinine Tiong et al. (2013), catharanthine, vindoline, vindolinene vinblastine, vincristine Bharti et al. (2018).
*Citrus x aurantium*	Narigin Pu et al. (2012), neohesperidin Osfor et al. (2013), Jia et al. (2015), epigallocatechin 3-gallate Chang et al. (2013b), diosmin, hesperetin Gothai et al. (2016), *p*-synerphine Suntar et al. (2018), N-acyl-2-aminothiazoles fused (+)-nootkatone Guo et al. (2020).
*Citrus limon*	Diosmin, eriodictyol, naringenin, hesperetin Gothai et al. (2016)
*Cola nitida*	caffein-rich Erukainure et al. (2017), caffeine and theobromine Erukainure et al. (2019).
*Cucumis sativus*	Kaempferol Ibitoye et al. (2017)
*Cyamopsis tetragonoloba*	Polyphenols-rich Gandhi et al. (2014)
*Erythrina abyssinica*	Benzofurans, coumestans Nguyen et al. (2010), flavonoids Ndinteh (2018)
*Eucalyptus globulus*	Euglobals, essential oils, macrocarpals Dey and Mitra (2013)
*Ficus exasperata*	α-amyrin acetate Nnamonu et al. (2016)
*Garcinia kola*	Kolaviron, a biflavonoid complex Adaramoye and Adeyemi (2006)
*Glycine max*	Daidzein, genistein, glycitein, beta-Sitosterol, Soyasaponin A1-A6, soyasaponin V, stigmasterol Tang et al. (2008), anthocyanins Nizamutdinova et al. (2009), lyceollin I-II Chang et al. (2013b), kaempferol glycoside rich fraction, kaempferol Zang et al. (2014), stigmasterol Wang et al. (2017b), soy isoflavones (genistein, diadzein) Bharti et al. (2018).
*Harungana madagascariensis*	Harunganols, kenganthranol A, harunganin, ferruginin A Johnson et al. (2015).
*Hibiscus esculentus*	Polysaccharide “rhamnogalacturonan” Liu et al. (2017).
*Jatropha curcas*	Flavonoid glycosides (rhiofolin, isoorientin, and isoquercetrin) El-baz et al. (2014)
*Lantana camara*	Stearoyl glucoside of ursolic acid (urs-12-en-3β-ol-28-oic acid 3β-D-glucopyranosyl-4′- octadecanoate) Kazmi et al. (2012)
*Mangifera indica*	The mangiferin Cruz-Vega et al. (2009), 1,2,3,4,6 penta-*O*-galloyl-β-d-glucose Mohan et al. (2013); curcumin, morin Gothai et al. (2016), gallic acid, 3,4-dihydroxy benzoic acid (Protocatechuic acid), kaempferol Ediriweera et al. (2017), flavonoids Pan et al. (2018); 1,2,3,4,6-penta-O-galloyl-β-D-glucoside, and 1,2,3,4,6-penta-O-galloyl-α-D-glucoside Yang et al. (2020).
*Momordica charantia*	Saponins Keller et al. (2011), cucurbitane triterpenoids Harinantenaina et al. (2006), Han et al. (2018), polysaccharide Xu et al. (2015), cucurbitane saponins Yue et al. (2017), saponins and polysaccharides Wang et al. (2019), insulin-like peptide, charantin, alkaloid vicine Pahlavani et al. (2019), 3β,7β,25-trihydroxycucurbita-5,23(E)-dien-19-al, charantal, charantoside XI, and 25ξ-isopropenylchole-5, 6-ene-3-*O*-D-glucopyranoside Shivanagoudra et al. (2019), polysaccharide-chromium (III) complex Zhang et al. (2019), saponins and polysaccharides Wang et al. (2019), momordicinin Kulkarni et al. (2021), Karaviloside VI and VIII Perera et al. (2021), 3β,7β,25-trihydroxycucurbita-5,23(*E*)-dien-19-al Noruddin et al. (2021), yeojoosides G-H, momordicoside U, karavilagenin A, goyaglycoside d, momordicoside F_1_, momordicoside L, momordicoside K, and 68 (3β,7β,23S)-3,7,23-trihydroxycucurbita-5,24-dien-19-al 7-β-D-glucopyranose Lee et al. (2021).
*Moringa oleifera*	Isothiocyanate-rich Waterman et al. (2016), protein (Mo-LPI) Paula et al. (2017), phenolic glycosides Wang et al. (2017b), 4-hydroxyphenylacetonitrite, fluoropyrazine, methyl-4-hydroxybenzoate, vanillin Hafizur et al. (2018)
*Musa x sapientum*	Rutin Kappel et al. (2013), syringin Sundaram et al. (2014)
*Ocimum gratissimum*	Chicoric acid Casanova et al. (2014)
*Olea europaea*	Oleuropein, oleanolic acid Sato et al. (2007), luteolin Dekdouk et al. (2015)
*Opuntia ficus-indica*	Polysaccharides El-mostafa et al. (2014), polyphenols, dietary minerals, betalains, gallic acid, vanillic acid, catechins Gupta et al. (2017), mucopolysaccharide Bharti et al. (2018)
*Oryza sativa*	γ-oryzanol Burlando and Cornara (2014), ferulic acid, *p*-coumaric Aalim et al. (2019), cyanidin 3-glucoside, and (2*R*,3*R*)-taxifolin Yoon et al. (2020).
*Phaseolus vulgaris*	Stigmasterol Tang et al. (2008), catechin Gothai et al. (2016), flavonoids and their glucosides of delphinidin, petunidin, and malvidin, anthocyanins, catechin, myricetin 3-O-arabinoside, epicatechin, vanillic acid, syringic acid, and O-coumaric acid Ganesan and Xu (2017), and triacylglycerols Sutedja et al. (2020).
*Phyllanthus amarus*	Oleanolic acid and ursolic acid (2:1) mixture Ali et al. (2006)
*Phyllanthus niruri*	Ellagic acid and its derivatives Bharti et al. (2018)
*Physalis angulata*	Withangulatin-A Raju and Mamidala (2015)
*Physalis peruviana*	Peruvioses A,B,C,D,E,F Bernal et al. (2018)
*Psidium guajava* L.	Quercetin, kaempferol, myricetin , Strictinin, isostrictinin Wang et al. (2010), pedunculagin, glycoprotein Chauhan et al. (2010); Singab et al. (2014), and polysaccharides Zhang et al. (2016).
*Pterocarpus marsupium*	Phenolic-C-glycosides Mishra et al. (2013)
*Punica granatum*	gallic acid Huang et al. (2005), valoneic acid dilactone Jain et al. (2012), ursolic and oleanolic Salah El Dine et al. (2014), polyphenols Tang et al. (2018), rutin, gallic acid, nictoflorin, and tulipanin El Deeb et al. (2021).
*Salvia officinalis*	Essential oils with 71.3% of monoterpenes Belhadj et al. (2018)
*Sesamum indicum*	(+)-Pinoresinol Wikul et al. (2012), furofuran lignans Worawalai et al. (2016)
*Solanum americanum*	Amide alkaloids Silva et al. (2017)
*Solanum melongena*	Phenylethyl cinnamides Liu et al. (2011)
*Spondias mombin*	3b-olean-12-en-3-yl (9Z)-hexadec-9-enoate Fred-Jaiyesimi et al. (2009)
*Syzygium cumini*	Gallic acid, umbelliferone, ellagic acid Perera et al. (2017), mallic acid, chlorogenic acid Bharti et al. (2018).
*Terminalia chebula*	Chebulagic acid Huang et al. (2012); Shyni et al. (2014), hydrolyzable tannins Lee et al. (2017).
*Trigonella foenum-graecum*	GII Puri et al. (2011), galactomannan Anwar et al. (2011), 4-hydroxyisoleucine Rangari et al. (2014); Naicker et al. (2016), diosgenin, galactomannan, flavonoids, trigonelline Zameer et al. (2017); isonarthogenin, 22β-acetoxyolean-12-ene- 3β, 24-diol, and soyasapogenol B Zhang et al. (2020).
*Urtica dioica*	Quercetin, quercetrin, apigenin, rutin, apigenin-7-*O*-glucoside Bharti et al. (2018).
*Vernonia amygdalina*	Sesquiterpenes Zhao et al. (2012), monoterpenes, sobrerol Li et al. (2013b), vernoamyoside E Anh et al. (2021)
*Vitis vinifera*	Cinnamic acid Guzman (2014), resveratrol, naringenin Gothai et al. (2016), proanthocyanidin, raisin Bharti et al. (2018).
*Xylopia aethiopica*	Oleanolic acid Mohammed et al. (2021).
*Zanthoxylum chalybeum *	Chaylbemides A-C, fagaramide, skimmianine, norchelerythrine, 6-acetonyldihydrochelerythrine, and 6-hydroxy-N-methyl decarine Ochieng et al. (2020).
*Zea mays*	Hirsutrin Kim et al. (2013), anthocyanins Hong et al. (2013), phenolics compounds Nile and Park (2014)
*Zingiber officinale*	Gingerol Sekiya et al. (2004), aframodial, camphene, 6-shogaol Tang et al. (2008), β-bisabolol Le (2014), 6-shogaol Fajrin et al. (2020).

To illustrate, *Allium cepa* contains ferulic acid, alliin (Tang et al., 2008), agavasaponin C (Tang et al., 2008), flavonoid alliuocide G (Mohamed, 2008), quercetin, sulfur compounds, S-methyl cystein sulfoxide, S-allyl cysteine and diallyl thiosulfanate (Bakhshaeshi et al., 2012; Noor et al., 2013). *Allium sativum* contains S-allylcysteine sulfoxide, alliin, diallyl trisulfide (Liu et al., 2007; Mikaili et al., 2013), isoeruboside B, agavasaponin C, proto-iso-erubisoide B, 2-Vinyl-4H-1,3-dithiin (Tang et al., 2008), allicin, diallyl disulfide, diallyl sulfide, ajoene, and allyl mercaptan (Bayan et al., 2014). In *mangifera indica*, one found mangiferin (Cruz-Vega et al., 2009), 1,2,3,4,6 Penta-O-galloyl-β-d-glucose (Mohan et al., 2013), curcumin, morin (Gothai et al., 2016), gallic acid, protocatechuic acid, kaempferol (Ediriweera et al., 2017). *Catarantus* has gallic acid, chlorogenic acid (Rianika and Robert, 2007), vindoline I, vindolidine II, vindolicine III and vindolinine (Tiong et al., 2013), catharanthine, vindoline, vindolinene, vinblastine, vincristine (Bharti et al., 2018). *Brassica juncea* (L.) Czern has cinnamic acid (Guzman, 2014), kaempferol (Gothai et al., 2016), aniline. *Bidens pilosa* has cytopiloyne, 2-β-D-Glucopyranosyloxy-1-hydroxytrideca-5,7,9,11-tetrayne (Chang et al., 2013a), polyynes (Bartolome et al., 2013), 3-β-D-glucopyranosyl-1-hydroxy-6(E)-tetradecene-8,10,12-triyne; 2-β-D-glucopyranosyloxy-1-hydroxy-5(E)-tridecene-7,9,11-triyne (Chang et al., 2013b). *Caesalpinia decapetala* has apigenin-7-rhamnoside, astragalin, 6-hydroxy kaempferol, quercitrin (Parveen et al., 2017). *Erythrina abyssinica* contains daidzein, genistein, glycitein, beta-Sitosterol, Soyasaponin A1-A6, soyasaponin V, stigmasterol (Tang et al., 2008), anthocyanins (Nizamutdinova et al., 2009), lyceollin I-II (Chang et al., 2013b), kaempferol glycoside, kaempferol (Zang et al., 2014), stigmasterol (Wang F. et al., 2017), genistein, diadzein) (Bharti et al., 2018). *Phaseolus vulgaris* contains stigmasterol (Tang et al., 2008), catechin (Gothai et al., 2016), flavonoids and their glucosides of delphinidin, petunidin, and malvidin, anthocyanins, catechin, myricetin 3-O-arabinoside, epicatechin, vanillic acid, syringic acid, and O-coumaric acid (Ganesan and Xu, 2017). *Tephrosia vogelii* has galactomannan (Anwar et al., 2011), 4-hydroxyisoleucine (Rangari et al., 2014; Naicker et al., 2016), diosgenin, galactomannan, flavonoids, trigonelline (Zameer et al., 2017). *Syzygium guineense* contains pinitol, β-sitosterol, quercetin, quercetin 3-O-α-L-rhamnopyranoside (Jawlal et al., 2013). *Aframomum melegueta* has 3 arylalkanes, 6-paradol, 6-shogaol, 6-gingerol, 6-gingeredione, a pentacyclic triterpene, oleanolic acid isolated from the fruit (Sugita et al., 2013; Mohammed et al., 2017).

#### 4.3.1 Alkaloids

The sulfur compounds present in the onion can significantly control the blood glucose and lipids in serum and tissues and normalize liver hexokinase, glucose 6-phosphatase and HMG CoA reductase (Akash et al., 2014). It was shown that vindoline I, vindolidine II, vindolicine III, and vindolinine improve the hyperglycemia condition of type 2 diabetes by enhancing glucose uptake in pancreatic or muscle cells. In addition, they can inhibit *in vitro* PTP-1B, which lessens insulin resistance. Vindolicine III was the most potent (Tiong et al., 2013). Catharanthine, vindolinene, vinblastine, vincristine lower blood sugar levels through free radical scavenging action (Bharti et al., 2018). On the other hand, p-synephrine increased the glucose output concentration and ameliorated glycolysis and glycogenolysis (Suntar et al., 2018). *N*-*trans*-*p-*coumaroyloctopamine, *N*-*trans*-*p-*feruloyl-octopamine, *N*-*trans*-*p*-coumaroyltyramine, and *N-trans*-*p*-feruloyltyramine, amide alkaloids, showed alpha-glucosidase effect and free radicals inhibitions (Silva et al., 2017).

#### 4.3.2 Amino Acids, Amines, and Carboxylic Acid Derivatives

Alliin offered protection against glucose or methylglyoxal-induced glycation of superoxide dismutase (Anwar and Younus, 2017). S-allyl cystein sulfoxide (SACS), allicin, and garlic oil precursor stimulated *in vitro* insulin secretion from beta cells isolated from normal rats (Kodera et al., 2017). It restored erectile function in diabetic rats (Yang et al., 2013). Unique and repeated intraperitoneal administrations of a protein (Mo-LPI) decreased blood glucose concentration at different times in rats. 2S, 3R,4S hydroxy isoleucine, an amino acid considered an insulinotropic agent, possesses antidiabetic potential by several mechanisms, including regulating glucose metabolism, lipid profile, and uric acid (Rangari et al., 2014).

#### 4.3.3 Carbohydrates and Sucrose Esters

Peruvioses A,B,C,D,E, and F possess antidiabetic potential by alpha-amylase inhibition activity (Bernal et al., 2018). In the Streptozotocin-induced diabetic mice group, rhamnogalacturonan (a polysaccharide) decreased blood glucose level and glucose tolerance and slightly improved blood glucose within 30 min (Liu et al., 2017). Polysaccharides repaired the pancreatic β cells damages in a high-fat diet STZ-induced type 2 diabetic mice by improvement of SOD concentration and the reduction of MDA level and restoration of kidney and pancreas tissues (Wang et al., 2019). Furthermore, a water-soluble polysaccharide significantly lowered fasting blood glucose level and improved glucose tolerance and weight loss in alloxan-induced diabetic mice compared to the diabetic control group (Xu et al., 2015).

#### 4.3.4 Glycosides

Cytopiloyne, a polyacetylene glucoside, reduced postprandial blood glucose levels, increased blood insulin, improved glucose tolerance, suppressed HbA1c level, and protected pancreatic islets in diabetic db/db mice (Chang et al., 2013b).

Supplementation of *Naringin* improved glucose intolerance and insulin resistance in a model of high-fat-diet–fed mice (Pu et al., 2012). Naringin (together with Neohesperidin, *h*esperidin, and nobiletin) significantly inhibited amylase-catalyzed starch digestion and played roles in hyperglycemia management by increasing hepatic glycolysis and glycogen concentration and lowering hepatic gluconeogenesis. Furthermore, hesperidin, naringin, and nobiletin reduced hepatic gluconeogenesis and improved insulin sensitivity in animal models (Lv et al., 2015). Neohesperidin significantly decreased fasting glucose, serum glucose, and glycosylated serum protein in mice. In addition, this compound significantly reduced serum triglycerides, total cholesterol, leptin level, and liver index; it inhibited lipid accumulation in the liver and decreased the size of epididymal adipocytes in the KK-Ay mice (Osfor et al., 2013; Jia et al., 2015).

Some phenolic glycosides, including niazirin A, S-Methyl-N-{4-[(α-l-rhamnosyloxy)benzyl]}thiocarbamate, reduced blood glucose levels in STZ-induced diabetic mice and promoted the glucose consumption of IR cells (Wang F. et al., 2017).

Isothiocyanates inhibited gluconeogenesis and hepatic glucose-6-phosphatase (G6P) expression in hepatoma cells and improved glucose tolerance and insulin signaling sensitivity (Waterman et al., 2016).

Galactomannan showed significant dose-related hypoglycaemic and antihyperglycaemic effects; the obtained results were better than glibenclamide used as reference (Anwar et al., 2011).

Aloe-emodin-8-*O*-glycoside enhanced glucose transport through proximal and distal marker modulation involved in glucose uptake and its transformation into glycogen (Salehi et al., 2018).

Syringin, a phenylpropanoid glucoside, indicated a significant reduction of blood glucose and HbA1c levels and improved transaminase enzymes, plasma protein, blood urea, serum creatinine, and uric acid levels. Inversely, it increased plasma insulin and hemoglobin levels in diabetic rats (Sundaram et al., 2014).

Rutin (a flavonol glycoside) significantly increased *in vivo* glucose-induced insulin secretion and acted as an insulin secretagogue in the management of glucose homeostasis (Kappel et al., 2013). Hirsutrin was suggested to prevent osmotic stress in hyperglycemia conditions by inhibiting RLAR activity and galactitol formation in rat lenses (Kim et al., 2013).

According to Fernando et al. (2019), three flavone C-glycosides, vicenin-1, isoschaftoside, and schaftoside, respectively, inhibited 60.3, 33.8, and 95.5% of pancreatic lipase enzyme, which plays a vital role in obesity (as a crucial factor in the occurrence of DMT2). Phenolic-C-glycosides enhanced and stimulated the glucose update process in mouse skeletal muscle cells (Mishra et al., 2013).

A stearoyl glucoside of ursolic acid (urs-12-en-3β-ol-28-oic acid 3β-D-glucopyranosyl-4′- octadecanoate) demonstrated an antidiabetic property by lowering sugar blood in rats from the 8th day to the 21st day of the experiment (Kazmi et al., 2012).

#### 4.3.5 Phytosterols

Sanni et al. (2019) suggested that sitosterol, stigmasterol, campesterol, squalene, and nimbiol might have antidiabetic potential through their molecular docking with AMP-activated protein kinase (α-AMPK) and alpha-amylase and alpha-glucosidase inhibitions. Stigmasterol increased GLUT4 translocation and expression *in vitro*. In mice, it alleviated insulin resistance, glucose tolerance by reducing fasting blood glucose levels and blood lipid (triglyceride and cholesterol) (Wang F. et al., 2017).

#### 4.3.6 Polyphenols

Quercetin and its glycosides protected β-cell mass and function under high-fructose induction (Li et al., 2013). 4'-methyl-quercetin-7-*O*-β-D-glucuronopyranoside enzymes, quercetin-3-*O*-glucoside, avicularin, castalagin, and 2,3-hexahydroxydiphenoyl-(α/β)-D-(4)C1-glucopyranose showed inhibition capacity of sucrase (Abdelhady et al., 2016). Moreover, they exhibited significant inhibition of alpha-glucosidase and alpha-amylase enzymes compared to acarbose (Wang et al., 2010; Olennikov and Kashchenko, 2014). A flavonoid named alliuocide G showed *in vitro* alpha-amylase inhibitory activity and radical scavenging potency (Mohamed, 2008).

Cinnamic acid and its derivatives (caffeic acid, ferulic acid, isoferulic acid, and *p*-hydroxycinnamic acid) are associated with a beneficial influence on Diabetes and its complications through many mechanisms. The most well-known are: stimulation of insulin secretion, improvement of pancreatic β-cell functionality, inhibition of hepatic gluconeogenesis, enhanced glucose uptake, increased insulin signaling pathway, delay of carbohydrate digestion and glucose absorption, and inhibition of protein glycation (Adisakwattana, 2017). Ferulic acid regenerated pancreatic beta-cells, reduced the risk of high-fat diet-induced hyperglycemia via insulin secretion and hepatic glucose-regulating enzyme activities, and regulated blood glucose levels by elevating glucokinase activity and production of glycogen (Silva and Batista, 2017). Caffeic acid produced a significant alpha-glucosidase inhibition comparing with acarbose (Olennikov and Kashchenko, 2014). In addition, together with chlorogenic acid and chicoric acid, it increased glucose uptake in muscle cells and stimulated insulin secretion from an insulin-sensitizing and insulin-secreting cell line and islets (Tousch et al., 2008; Ferrare et al., 2017). Caffeoylquinic acid derived from caffeic acid showed high inhibitory activity against digestive enzymes, exceptionally higher against alpha-amylase and alpha-glucosidase (Olennikov et al., 2018).

A study indicated that epigallocatechin and epigallocatechin gallate reduced fasting blood glucose levels, triglycerides, and total cholesterol in streptozotocin-induced diabetic mice (Bakoma et al., 2018). Also, apigenin-7-rhamnoside, astragalin, 6-hydroxy kaempferol, quercitrin exhibited significant activity against alpha-glucosidase enzyme (Parveen et al., 2017). Kaempferol (Fraction B) lowered blood glucose of alloxan-induced diabetic rats. It also inhibited alpha-amylase and alpha-glucosidase and reversed altered lipid profile and oxidative stress biomarkers in diabetic rats (Ibitoye et al., 2017). Kaempferol and myricetin showed high inhibitory activities against alpha-amylase and alpha-glucosidase (Wang et al., 2010).

Compared with the reference compound acarbose, Aesculetin and isorhamnetin demonstrated significantly higher inhibitory activity (Olennikov and Kashchenko, 2014). Polyphenols compounds such as proanthocyanidins and anthocyanins showed as potential natural alpha-glucosidase inhibitors (Dey and Mitra, 2013).

Anthocyanins efficiently protected pancreatic beta-cells from cell death in HIT-T15 cell culture and db/db mice (Hong et al., 2013). Johnson et al. (2015) demonstrated that prenylated anthranols possess an alpha-glucosidase inhibitory potential. According to their findings, the most antidiabetic activity was found with harunganol compared to acarbose.

Kolaviron, a bioflavonoid complex, demonstrated a significant reduction of glycemia in normoglycemic rats. Moreover, kolaviron showed a significant antidiabetic potential in streptozotocin-induced rats (Adaramoye and Adeyemi, 2006).

Ellagic acid and its derivatives act as a hypoglycaemic agent on carbohydrate digestion and absorption, insulin secretion (Bharti et al., 2018). Hydrolyzable tannins including 1,2,3,6-tetra-*O*-galloyl-4-*O*-cinnamoyl-b-D-glucose and 4-*O*-(200,400-di-*O*-gal-loyl-a-L-rhamnosyl) ellagic acid showed significant alpha-glucosidase inhibitory efficacy with IC_50_ values of 2.9 and 6.4 mM, respectively (Lee et al., 2017).

Chicoric acid lowered the glycaemic levels of diabetic mice (Casanova et al., 2014) significantly. Valoneic acid dilactone, a hydrolyzable tannin, showed a potential antidiabetic effect alpha-amylase enzyme activity compared to the value obtained by acarbose. In the same way, it significantly inhibited aldose reductase enzyme activity and PTP1B enzyme activity. However, *in vivo* evaluation, it reduced the BGL considerably in acute evaluation for 4 h. Furthermore, oral administration of the compound for 21 days significantly decreased BGL and improved the tolerance to glucose compared to control groups (Jain et al., 2012).

Gingerols demonstrated antidiabetic potential by enhancing glucose uptake. Primarily, (S)-[8]-Gingerol was found to be the most potent on glucose uptake and increase in the surface distribution of GLUT4 protein on the L6 myotube plasma membrane (Noipha and Ninla-aesong, 2018).

*ρ*-Coumaric acid exhibited higher inhibition activity against alpha-glucosidase (98.8%) than acarbose (62.5%). However, acarbose showed the most potent inhibition against alpha-amylase (98.6 vs 66.8%) (Aalim et al., 2019).

Luteolin showed significant alpha-glucosidase and alpha-amylase inhibitory activities (Dekdouk et al., 2015). Chebulagic acid (a benzopyran tannin) reduced maltose-hydrolysis and sucrose-hydrolysis activities. Meanwhile, it induced a decrease at 11.1% of postprandial blood sugar value in maltose-loaded Sprague-Dawley rats (Huang et al., 2012).

Furofuran lignans with a free hydroxyl synthesized from herein demonstrated an inhibition potential against alpha-glucosidase and free radicals (Worawalai et al., 2016). Previously, Wikul et al. (2012) conducted bio-guided isolation and showed that (+)-pinoresinol, a lignan, had inhibitory activity against rat intestinal maltase. Also, *N*-*trans*-feruloyl tyramine, *N*-*trans*-*p*-coumaroyl tyramine, and *N*-*cis*-*p*-coumaroyl tyramine (Phenylmethyl cinnamates) showed inhibitory activity against alpha-glucosidase (Liu et al., 2011).

#### 4.3.7 Saponins

Pseudoprototinosaponin AIII and prototinosaponins AIII produced a hypoglycaemic effect on glucose uptake and insulin release due to their actions on hepatic gluconeogenesis or glycogenolysis (Patel et al., 2012). Furostanol saponin showed significant antidiabetic potential *in vitro* by reducing the fasting plasma glucose level by 46.14% and increasing insulin and C-peptide levels (Ezzat et al., 2017). [3β,7β,25- trihydroxycucurbita-5,23(E)-dien-19-al, momordicine I, momordicine II, 3- hydroxycucurbita-5,24-dien-19-al-7,23-di-*O*-β-glucopyranoside, and kuguaglycoside G] were potent in the β-cell insulin secretion evaluation. Momordicine II and kuguaglycoside have stimulated insulin secretion 7.3 and 7.1 times and 8.1 and 7.8 times more, respectively, than the control group (Keller et al., 2011).

25-O-methylkaraviagein D, karaviloside II, and (19R,23E)-5b,19-epox y-19,25-dimethoxycucurbita-6,23-dien-3b-ol, cucurbitane exhibited significant inhibitory activity on alpha-glucosidase with IC_50_ values of 10.19, 28.55, and 20.20 µM, respectively (Yue et al., 2017). Oral administration of saponins improved body weight and insulin resistance. There was an increase in fasting blood glucose concentration and the proportion of hepatic phosphorylated adenosine monophosphate-activated protein kinase (p-AMPK)/total protein (Wang et al., 2019).

#### 4.3.8 Terpenoids

Oleanolic acid, a plant-derived triterpenoid, boosted insulin secretion *in vitro* and stimulated insulin secretion at both basal and stimulatory glucose concentrations in INS-1 832/13 cells, and enhanced acute glucose-stimulated insulin secretion cultured β-cells (Teodoro et al., 2008). Furthermore, it decreased serum glucose and insulin concentrations in mice fed with a high-fat diet and enhanced glucose tolerance (Sato et al., 2007). Oleanolic and ursolic acids showed potent alpha-glucosidase and alpha-amylase inhibition. Ursolic acid showed uncompetitive inhibition of alpha-glucosidase compared to acarbose as a competitive inhibitor (Ali et al., 2006; Salah El Dine et al., 2014).

Thujone, a monoterpene existing as two stereoisomers (α- and β-Thujone), is an ingredient of essential oils of many great different herbs; it can increase free insulin-stimulated glucose transporter by activation of adenosine monophosphate-activated protein kinase (Daradka et al., 2014).

α-amyrin acetate (a pentacyclic triterpenoid) lowered the blood glucose profile in STZ-induced diabetic rats and db/db mice at 50 mg kg1 dose level (Singh et al., 2009). Pahlavani et al. (2019) showed that some compounds like charantin (a triterpenoid phytoconstituent), possess antidiabetic potential by several mechanisms, including insulin secretion increase, insulin resistance decrease, skeletal muscle cell glucose utilization increase, and inhibition of intestinal enzymes.

Cucurbitane-type compounds (3β,7β,25-trihydroxycucurbita-5,23(E)-dien-19-al, charantal, charantoside XI, and 25ξ-isopropenylchole-5, 6-ene-3-O-D-glucopyranoside), demonstrated an alpha-amylase and alpha-glucosidase inhibitory activities ranging from 56 to 79% (Shivanagoudra et al., 2019).

Two monoterpenes (1S,2R,3R,5S)-2-hydroxymethyl-6,6-dimethylbicyclo[3.1.1]heptane-2,3- diol, and sobrerol significantly increased glucose uptake in 3T3-L1 adipocytes (Li et al., 2013b). On the other hand, three germacrene sesquiterpenes increased glucose uptake substantially without significant toxic effects in 3T3-L1 adipocytes (Zhao et al., 2012).

## 5 Conclusion

Multiple investigations have been carried out on natural products, mainly plants used to treat Diabetes Mellitus worldwide. In DRC, a country with a high ecological, cultural and human diversity, traditional medicine through plants occupies an important place in the health system. Several ethnopharmacological and ethnobotanical studies have been conducted previously in this perspective, and various plant species have been identified. Contrary to the previous review, the present review assessed the quality of studies carried inside DRC and resorted similarities/discrepancies with studies conducted outside. The findings confirm the high diversity of the flora and the various ethnic groups in DRC. Most of the plants claimed as antidiabetic and used by traditional healers in the DRC are not specifically native to DRC. One hundred thirty-four native and introduced species have been experimentally validated by various pharmacological, toxicological, and phytochemical researches. Many plants are safe at doses < 500 mg/kg, but long-term use may trigger sub-chronic toxicity. Exclusively conducted in DRC, preclinical and clinical studies of some plant species demonstrated poor protocol quality. Locally specific species deserve in-depth investigations to meet scientific requirements for their introduction studies into the national pharmacopeia. Although a few plants reduced blood sugar levels, clinical data and antidiabetic studies of the isolated compounds remain limited to allow the availability and accessibility of standardized phytomedicines to Congolese. This review constitutes a primary database for further experimental studies, especially for unstudied species in the perspective of safe and efficient use of easily accessible natural resources.

## Abbreviations

11ß-HSD1, 11β-hydroxysteroid dehydrogenase type 1; BG, blood glucose; DM, diabetes mellitus; DMT2, Diabetes mellitus type 2; DPP-4, dipeptidyl peptidase-4; DRC, Democratic Republic of Congo; HIV/AIDS, human immunodeficiency *virus/*Acquired immunodeficiency syndrome; GLP-1, glucagon-like peptide- 1; GLUT, Glucose Transport; HbA1c, Glycosylated hemoglobin; LD_50_, lethal dose 50; mRNA, Messenger ribonucleic acid; OGTT, Oral Glucose Tolerance Test; PPAR, peroxisome proliferator-activated receptor; PTP1B, Protein tyrosine phosphatase 1B.
